# Multifunctional electrospun PCL/CNT/EGCG nerve conduits with a collagen hydrogel for enhanced sciatic nerve regeneration

**DOI:** 10.1186/s12967-025-07561-5

**Published:** 2025-12-23

**Authors:** Fahimeh Ahmadi, Elham Hasanzadeh, Amir Mellati, Roya Fattahi, Ali Reza Khalatbary, Pedram Ebrahimnejad, Narges Karimi, Mozhgan Abasi

**Affiliations:** 1 Student Research Committee, School of Advanced Technologies in Medicine, Mazandaran University of Medical Sciences, Sari, Iran; 2 Immunogenetics Research Center, Faculty of Medicine, Mazandaran University of Medical Sciences, Sari, Iran; 3 Department of Tissue Engineering & Regenerative Medicine, School of Advanced Technologies in Medicine, Mazandaran University of Medical Sciences, Sari, Iran; 4 Molecular and Cell Biology Research Center, Faculty of Medicine, Mazandaran University of Medical Sciences, Sari, Iran; 5 Department of Pharmaceutics, Faculty of Pharmacy, Mazandaran University of Medical Sciences, Sari, Iran; 6 Department of Neurology, School of Medicine, Immunogenetic Research Center, Mazandaran University of Medical Sciences, Sari, Iran

**Keywords:** Nerve guidance conduits (NGCs), Polycaprolactone (PCL), Epigallocatechin gallate (EGCG), Multiwall carbon nanotubes (CNTs), Electrospinning, Tissue engineering, Peripheral nerve regeneration

## Abstract

**Background:**

Damage to peripheral nerves results in sensory, motor, and autonomic impairments, which are frequently accompanied by neuropathic pain. Effective strategies for nerve regeneration remain a major clinical challenge.

**Methods:**

We engineered nanofibrous nerve guidance conduits (NGCs) composed of poly(ε-caprolactone) (PCL), multiwalled carbon nanotubes (CNTs), and epigallocatechin gallate (EGCG), which were fabricated by electrospinning and subsequently filled with a collagen hydrogel. CNTs were incorporated to improve the mechanical strength, physicochemical properties, and electrical conductivity, whereas EGCG had anti-inflammatory and antioxidant effects. The scaffold morphology was evaluated by scanning electron microscopy (SEM) and atomic force microscopy (AFM). In vitro assays were used to assess mesenchymal stem cell (MSC) viability and morphology. Functional recovery was examined via sciatic nerve functional indices and gastrocnemius electromyography.

**Results:**

At 12 weeks post-axotomy, stereological, immunohistochemical, MRI, and real-time PCR analyses of sciatic nerves and L4–L5 dorsal root ganglia revealed significant upregulation of neuronal markers (MAP2, β-tubulin III, and neurofilament) and Schwann cell markers (S100, and NF-200), suggesting enhanced neuronal maturation and myelination. Compared with the control conditions, the composite conduits promoted improved motor function, nerve conduction velocity, and muscle preservation.

**Conclusion:**

PCL/CNT/EGCG nanofibrous conduits, combined with a collagen hydrogel provide a favorable microenvironment for peripheral nerve regeneration. This strategy has translational potential for improving outcomes following peripheral nerve injury.

**Graphical abstract:**

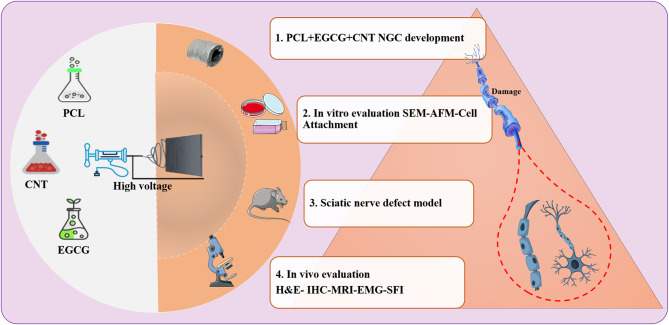

## Introduction

Injury to peripheral nerves is a significant biomedical concern that can severely impair the quality of health-related life. While peripheral nerves can regenerate following a lesion, this process often fails to achieve full functional recovery. Despite an extensive understanding of regeneration mechanisms, treatments that guarantee complete functional restoration are rare. A variety of factors, including the patient’s age, the specific nerve affected, the site and extent of the injury, the damaged nerve trunk, the surgical repair technique, the span of the nerve defect, and the time period following damage and therapy, influence effective functional recovery [1–3]. According to Seddon, nerve injuries can be divided into three classifications, taking into account the presence of demyelination and the severity of damage to both axonal structures and the connective tissues within the nerves [4, 5]. Neurapraxia is characterized by localized demyelination without any damage to axonal structures and usually arises from minor mechanical compression or tensile stress on the nerve. This results in a reduced conduction velocity, with effects varying from irregular nerve signals to complete signal blockage, resulting in muscle weakness. Axonotmesis represents the next level of nerve injury and is characterized by axonal damage and focal loss of the myelin sheath; however, the connective tissue framework of the nerve remains intact. The most intense form, neurotmesis, incorporates a comprehensive severance of axonal and connective tissue components, resulting in total nerve discontinuity [6, 7]. The distance between the severed nerve ends is a key factor in determining the success of nerve repair. In humans, gaps of 4 cm have a good chance of healing, whereas gaps > 4 cm usually show minimal or no healing. This limitation is due to the mechanical and biological challenges associated with axonal growth over long distances. Compensatory mechanisms such as lateral sprouting (the growth of healthy axons into denervated areas) also have limited efficacy. This process often results in incomplete restoration of sensorimotor functions, particularly in larger neural pathways that handle fine motor control and peripheral sensitivity [8].

Regenerating axons must be enclosed by the neurilemma and endoneurium sheaths to support targeted guidance. The proximal and distal ends of the injured nerve must be aligned in the same anatomical plane to prevent neuroma formation (aberrant growth of nerve tissue [9–11]).

Owing to their critical size, limited capacity for spontaneous regeneration, and lack of complete functional recovery, peripheral nerve injuries represent a major clinical challenge. Conventional therapies such as autografts have limitations, resulting in poor outcomes due to donor site complications, limited accessibility, architectural mismatch, and inadequate bioactivity [12].

Tissue engineering presents an emerging approach to address the constraints of classical treatments and the results of tissue restoration. By incorporating engineered constructs into damaged nerve zones, the rehabilitation and operational restoration of neural disruptions can be substantially improved [13–15]. Recent studies have explored various polymeric nerve guidance conduits (NGCs) with promising outcomes. For example, Sun et al. (2024) developed an electrically aligned polyurethane conduit that modulated macrophage polarization and enhanced immunoregulatory regeneration [16]. Redolfi Riva et al. (2024) investigated implantable NGCs for bridging long nerve gaps, although their design lacked dynamic bioactivity and long-term integration [17]. Lam et al. (2024) reviewed advanced biomaterials for neuroregeneration, highlighting electroactive and stem-cell compatible scaffolds, but noted limitations in sustained bioactive molecule delivery and immune modulation [18]. Despite the progress made in recent years, the use of antioxidant, immunomodulatory, and conductive elements in a single biodegradable conduit still has some unresolved issues for clinical use [19]. In this composite system, PCL and CNTs form a mechanically strong and electroactive scaffold that supports nerve signal conduction, whereas collagen enhances cell adhesion and differentiation. The incorporation of EGCG suppresses the NF-κB and MAPK pathways, reducing cytokine secretion and oxidative stress, thereby preventing inflammation and glial scar formation and promoting a stable microenvironment for nerve regeneration. In this study, a multifunctional scaffold was created by combining PCL nanofibers for mechanical reinforcement of the conduit, EGCG as a potent anti-inflammatory antioxidant with neurotrophic effects, and a collagen hydrogel as a physical and biological substrate for nerve growth and an EGCG carrier.

A suitable tissue scaffold must encompass an interactive microenvironment through biological processes with topographical precision. Recently, the creation of bioactive conduits for the transmission of nerve impulses has presented a potential substitute for autografts. Various materials (collagen, laminin, gelatin, and chitosan), such as extracellular matrix components, recombinant protein fusions, exosome complexes, and transplanted cells that secrete mitogenic factors, have been used in animal studies to evaluate their effectiveness in neural tube formation. [14, 15, 20]. Nonetheless, the use of artificially produced materials has become increasingly prevalent in nerve repair owing to certain inherent limitations derived from biobased materials. Notable examples of synthetic biomaterials include polycaprolactone (PCL), polyurethane (PU), polylactic acid (PLA), and poly(lactic-co-glycolic acid) (PLGA) [21, 22].

Synthetic polyesters, particularly PCL nanofibrous scaffolds with diverse structural arrangements, increase neurite outgrowth but require the incorporation of bioactive molecules (e.g., laminin, fibronectin and nerve growth factor) for successful nerve regeneration. The use of electrospun nanofibers with nanostructured fillers improved the mechanical properties and degradability of PCL. This polymer is a semicrystalline linear polyester with a crystallinity of up to 69%, which is approved by the Food and Drug Administration (FDA) for medical applications. In terms of degradability, it degrades to lactic acid upon hydrolysis, and the degradation period lasts from several months to several years (depending on molecular weight and environmental conditions) [23].

PCL scaffolds enhance the architecture of the structure by mimicking the natural extracellular matrix with a high surface area and tunable mechanical properties. Therefore, it is a prominent option for central nervous system (CNS) and peripheral nervous system (PNS) repair [24–26].

Multiwall carbon nanotubes (CNTs) have been identified as particularly advantageous materials because they significantly increase the transmission of neuronal signals, aid in the elongation of dendrites, and promote cell adhesion [27]. Numerous studies have confirmed that CNTs serve as highly efficient electrical conductors within scaffolds and are capable of influencing both the impulse transmission and spatial configuration of neurons, which significantly affects their proliferation and specialization processes. [28] Additionally, the inherent high porosity and biocompatibility of these materials ensure that they are nontoxic and safe for cells [29, 30].

**Fig. 1:**
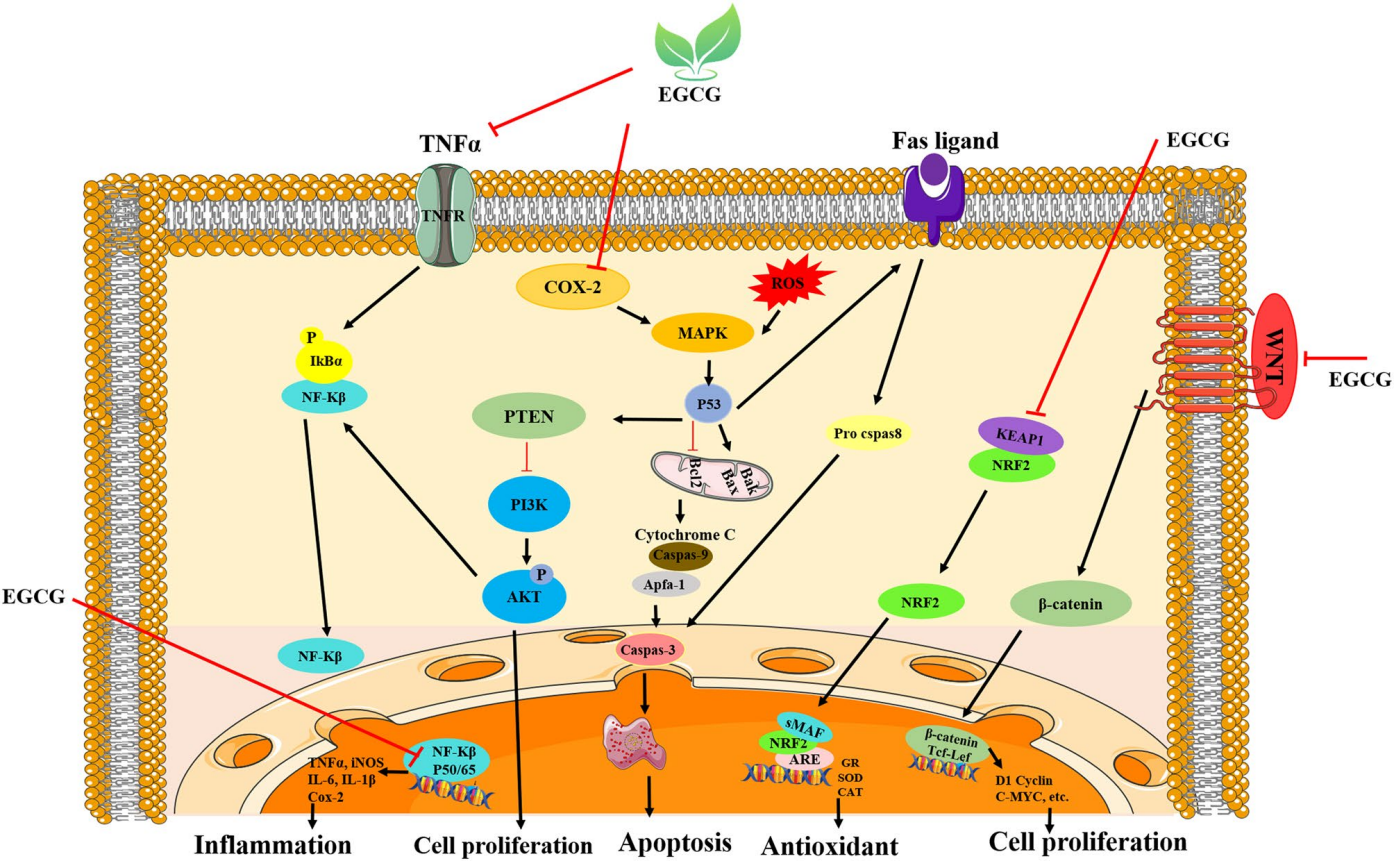
Regulation of key cellular signaling pathways by EGCG. Schematic representation of the biological effects of EGCG on membrane receptors (TNFR, fas ligand, and laminin receptor) and intracellular kinases. EGCG inhibits NF-κB, COX-2, MAPK, and PI3K/AKT signaling to suppress inflammation and abnormal cell proliferation; activates p53 and caspase cascades to induce apoptosis; and enhances antioxidant properties through stimulation of the Nrf2/ARE pathway, thereby contributing to cellular homeostasis and protection against oxidative stress

Green tea has garnered global interest for its extensive health benefits and abundance of flavonoids and catechins [31]. These compounds play crucial roles in neutralizing oxygen-derived free radicals in the human body. Among the diverse array of polyphenolic components, epigallocatechin gallate (EGCG) is recognized as a highly effective potent scavenger of unstable oxygen-derived radicals [32]. EGCG effectively scavenges reactive oxygen species (ROS), thereby reducing oxidative stress and preserving neuronal integrity. It modulates key intracellular signaling pathways, including the inhibition of proapoptotic cascades, such as JNK and ASK-1, while simultaneously activating survival-promoting pathways, such as PKC, and upregulating antiapoptotic proteins, such as Bcl-2. EGCG also suppresses neuroinflammation by downregulating NF-κB and proinflammatory cytokines (e.g., TNF-α and IL-1β), contributing to a more favorable microenvironment for neuronal survival (Fig. 1). EGCG delivery in amounts equivalent to 25 or 50 mg/kg considerably reduces oxidative stress in motor neurons [33]. Furthermore, EGCG enhances the nuclear factor erythroid 2-related factor 2 (Nrf2)-dependent antioxidant mechanism, serving as a key element in scavenging oxidative agents in human cells [34]. To investigate the role of EGCG in modulating stress from free radicals and associated immune responses in the context of peripheral neuropathy, scaffold-based controlled release systems allow for gradual and localized drug delivery, which increases long-term improvement due to the limitations of daily injections [35, 36]. Collagen, a major structural component of nerves and other tissues, is essential for maintaining tissue integrity and supporting the formation of a cohesive extracellular matrix. Collagen-containing channels are semipermeable and biocompatible [37]. Recent studies have shown that when the mechanical properties and degradation rate of a scaffold do not match those of the surrounding tissue, it can cause inflammation and fibrosis. This emphasizes the importance of designing multilayer structures with appropriate biomechanical behavior to minimize these reactions [38]. Additionally, the use of a combination of natural and synthetic materials in nerve conduction channels, along with the use of coatings or anti-inflammatory agents, can influence the immune response and reduce chronic inflammation [39]. Another study showed that EGCG-loaded scaffolds improved axonal growth and function in a rat model of sciatic nerve injury [40]. These findings suggest that EGCG may have therapeutic potential for peripheral nerve injury. However, current strategies still have shortcomings [19]. In this study, we developed electrospun NGCs composed of PCL, CNTs, and EGCG filled with a collagen hydrogel to promote sciatic nerve regeneration. This design enables the controlled and localized release of EGCG, with the aim of mitigating inflammation and oxidative stress at the injury site. We hypothesize that the synergistic integration of structural support (PCL), electrical conductivity (CNT), and sustained antioxidant delivery (EGCG) will enhance neural repair and functional recovery in peripheral nerve injuries.

## Materials and methods

### Materials

The substances used were EGCG and PCL (molecular weight of 6000, Sigma‒Aldrich USA); carbon nanotubes (CNTs) (functionalized with carboxyl groups (CNT-COOH), purity greater than 95%, outer diameter of 20–30 nm, from US NANO (USA); Dulbecco’s modified Eagle’s medium(DMEM/F12, (US4302, Invitrogen, USA); fetal bovine blood serum (FBS 10,270–106, Gibco, USA); penicillin/streptomycin (15070, Gibco, USA); trypsin-EDTA (25300–054, Gibco, USA); chloroform and methanol (Merck, German); phosphate-buffered saline (PBS, Sigma_Aldrich Germany); 3-(4, 5-dimethylthiazol-2-yl)-2, 5-diphenyl tetrazolium bromide (MTT, M2128, Sigma_Aldrich, Germany); and 4′, 6-diamidino-2-phenylindole (DAPI, Sigma_Aldrich, Germany).

### Formulation of solutions for the spinning processes

**Fig. 2:**
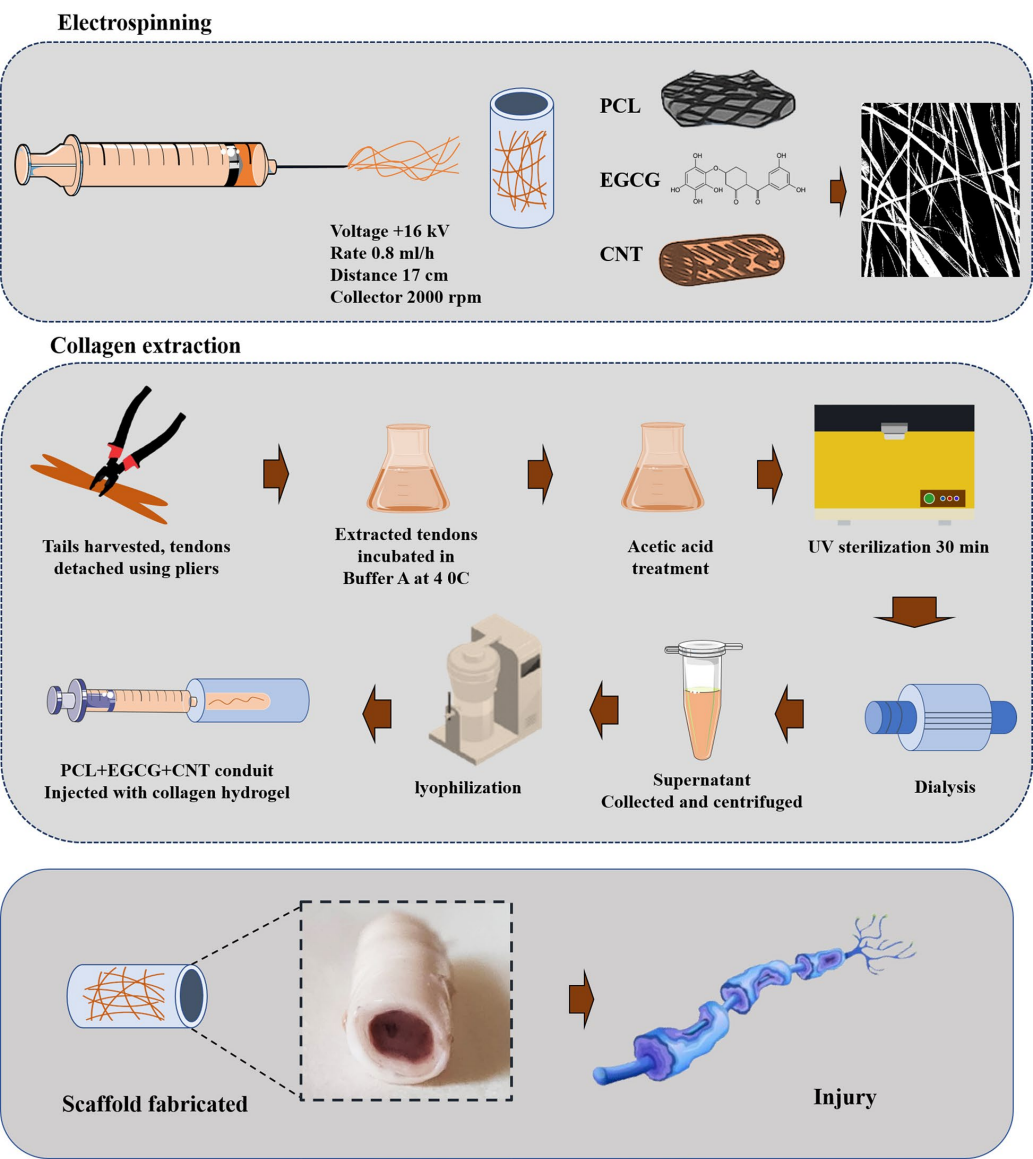
Schematic overview of scaffold fabrication for tendon tissue engineering. The process involves three sequential stages: (1) *electrospinning* of a composite solution containing PCL, EGCG, and CNTs under optimized parameters (16 kV, 0.8 mL/h, 17 cm, 2000 rpm) to produce aligned nanofibers; (2) *collagen extraction* from rat tail tendons via buffer incubation, acetic acid treatment, UV sterilization, and dialysis; and (3) *scaffold* assembly by injecting a collagen hydrogel into the electrospun conduit followed by lyophilization, yielding a bioactive scaffold suitable for nerve regeneration

The solution contained 12% (w/v) PCL, 1% EGCG, and 0.25% (w/v) CNTs, and the preparation of the 12% (w/v) PCL formulation involved dissolving 0.30 g of PCL in 2.5 ml of a chloroform/methanol (70/30 ratio) solvent mixture. To ensure homogeneity, EGCG was then dissolved in the same chloroform and methanol (70/30) solvent composition at 37 °C for one hour while being stirred magnetically. The preparation of the 0.25% (w/v) CNT–COOH suspension involved dispersing the compound via bath sonication for 3 to 4 h (Fig. 2).

### Fabrication of NGCs through electrospinning

In the process of spinning, the requisite chemicals and solvents were precisely measured and combined. PCL was dissolved in chloroform and methanol to achieve the desired concentration. CNT-COOH and EGCG were subsequently added to the solution. The mixture was then subjected to ultrasonication to facilitate even dispersion of the nanoparticles. Continuous stirring at standard temperature was sustained until a uniform solution was produced, ensuring the complete dissolution of all the constituents.

A single electrospinning nozzle was used to fabricate the PCL+EGCG+CNT mixture nanofiber film. The solution comprising PCL, EGCG, and CNTs was loaded into a 5 ml syringe equipped with an 18 G needle, which was subsequently connected to a high-voltage electrical source. The extruded solution was deposited onto a sheet of aluminum foil at ambient temperature. To achieve oriented nanofibers, the drum was rotated at a speed of 2000 rpm. The parameters for the injection of the PCL solution were as follows: a voltage of 16 kV, a spinning rate of 0.8 ml/h, and a distance of 17 cm between the needle and the collector at a speed of 2000 rpm.

Following the electrospinning process, the samples were immersed in distilled water for 10 min to ensure the complete removal of any residual toxic solvents. The PCL+EGCG+CNT nanofibrous mats were carefully removed from the aluminum foil and wrapped tightly around a stainless-steel rod with a diameter of 2 mm. The scaffolds were subsequently sterilized via UV light for 30 min (Fig. 2).

### Collagen extraction and NGC filling

Type I collagen was extracted from rat tail tendons via a standard acid dissolution protocol. Tendons were dissected from the vertebrae via sterile forceps and incubated in buffer A at 4 °C. The tissues were then treated with 0.1% acetic acid, and the resulting supernatant was centrifuged to remove insoluble components. The collagen-rich solution was dialyzed and lyophilized for further use [41, 42] (Fig. 2). For hydrogel synthesis, 0.4% lyophilized collagen was dissolved in 0.1% acetic acid. The solution was neutralized with phosphate-buffered saline (PBS) and 0.1 normal NaOH (pH = 7.4). The mixture was then incubated at 37 °C for 30–60 min to initiate gelation. The heat-induced gelation process allows self-assembled collagen molecules into a fibrous network resembling natural connective tissue. No chemical crosslinkers were added to maintain bioactivity. The resulting hydrogel exhibited a semigel consistency with uniform density. Scanning electron microscopy (SEM) revealed an average pore size of 80–100 μm, which was chosen to match the typical diameter of peripheral neurons (10–40 μm) and facilitate cellular penetration, axonal extension, and nutrient diffusion [43] (Fig. 2).

### Characterization of the fabricated nanofibers

To investigate the shape of the electrospun nanofibers, both scanning electron microscopy (SEM) and atomic force microscopy (AFM) were used. SEM provided a detailed 3D surface morphology, revealing the structural and alignment characteristics of the nanofibers. Moreover, AFM allows for 3D topographical analysis, offering insights into surface roughness and texture at the nanoscale [44].

#### Atomic force microscope (AFM)

The characterization of the nanofibers, including PCL, PCL+EGCG, PCL+CNT, and PCL+EGCG+CNT, for AFM testing was conducted via an MF-3D atomic force microscope from Instruments in Oxford, England. The microscope was operated at a scanning rate of 1 Hz, and the dimensions of the scanning area varied between 10 × 10 μm and 20 × 20 μm.

#### SEM analysis

To investigate the surface features and diameter of the PCL, PCL+EGCG, PCL+CNT, and PCL+EGCG+CNT nanofibers, a digital vacuum scanning electron microscope (AIS2300C, Korea) operating at an electron beam voltage of 15 kV was used. The nanofiber surfaces were sputter-coated with gold prior to characterization by scanning electron microscopy. The average diameter of the nanofibers was determined via SEM analysis of 100 randomly selected fibers (*n* = 6) via ImageJ software (version 1.52).

#### Infrared spectroscopy via Fourier transform (FTIR)

The chemical, functional, and potential interactions among the components of the composite scaffolds were evaluated via FTIR with a Cary630-Agilent instrument from the USA. The analysis was executed over a scan range of 500–4000 cm−1, with the resolution set at 4 cm−1.

#### Mechanical property measurements

A general testing apparatus (STM-5, Santam Co., Tehran, Iran) was utilized to assess the mechanical properties of the conduits. For this evaluation, three samples of each variant—PCL, PCL+EGCG, PCL+CNT, and PCL+EGCG+CNT—were fabricated. Each sample was shaped into a rectangle measuring 5 mm in width and 20 mm in length. Tensile testing was performed under a load of 70 N and an extension rate of 1 mm/min. The standard deviations (SDs) of the measured values were documented, as were the ultimate tensile strength and the lengthening at rupture. The ultimate tensile strength (*UTS*), Young’s modulus (*E*), and elongation at break (Ɛ*b*) were extracted from the stress‒strain curves recorded throughout the tensile tests.

#### Four- point probe technique

The electrical conductance of electrospun PCL, PCL+CNT, and PCL+EGCG+CNT nanofibers (*n* = 3) was assessed via a four-point probe technique (a Signatone SYS-301 in conjunction with a Keithley 196 system DDM multimeter). Measurements were conducted both before and after the incorporation of the MWCNTs into the electrospun PCL nanofibers. This process involved applying a voltage to a square-shaped sample and subsequently recording the resulting electrical current.

#### Contact angle test

To assess the hydrophobic properties of the synthesized nanofilaments, the water contact angle was measured at three distinct time intervals for each type of test piece, namely, PCL, PCL+EGCG, PCL+CNT, and PCL+EGCG+CNT nanofibers. This measurement was conducted via a static contact angle goniometer (KRUSS, Hamburg, Germany). At ambient temperature, a 5 μl droplet of deionized water was placed on the surfaces of the fibers affixed to coverslips. The tangent line at the contact point between the water droplet and the fiber surface was recorded within 10 s of droplet application.

#### Assessment of the degradation rate and pH fluctuation

To examine the in vitro degradation of the PCL, PCL+EGCG, PCL+CNT, and PCL+EGCG+CNT conduits, each sample was placed in 5 mL of PBS with a pH of 7.4 at a controlled temperature of 37 °C. At predetermined time intervals (weeks 1, 2, 4, 6, and 8), three samples were removed from the PBS, washed with distilled water, air dried, and weighed to quantify the degradation level. The weight loss was computed via the following equation. In this equation, *Wt* represents the initial weight of the conduit, and *W1* signifies the weight after drying after extraction from the PBS. The average weight loss for each conduit was derived from three measurements:

1 $$Weight{\text{ }}loss{\text{ }}\left( \% \right){\text{ }} = {\text{ }}Wt - W1/Wt{\text{ }} \times 100$$

In addition, the changes in pH during the degradation of each sample were recorded at each designated time point (weeks 1, 2, 4, 6, and 8) via a digital pH meter (ZAG CHEME CO Model PTR79).

#### Equilibrium swelling ratio

PCL, PCL+EGCG, PCL+CNT, and PCL+EGCG+CNT conduits of known weights were incubated in a PBS solution at 37 °C for 48 hours, allowing them to reach swelling equilibrium. At specified time points (*n* = 3), the expanded fibers were extracted from the solution, and any excess water was eliminated with tissue paper. The samples were weighed with a microbalance. The swelling index was calculated via eq. (2), where *Ws* represents the weight of the sample after swelling stabilization and *Wi* represents the initial weight of the sample.

2 $$Swelling\,test{\text{ }} = {\text{ }}Ws - Wi/Wj{\text{ }} \times 100$$

#### Measurement of porosity

The evaluation of porosity in the conduits was carried out via the liquid displacement method, incorporating the equation provided below (3).

3 $$Porosity{\text{ }}\left( \% \right){\text{ }} = {\text{ }}v1{\text{ }} - {\text{ }}v3/v2{\text{ }} - {\text{ }}v3{\text{ }} \times {\text{ }}100$$

Following the immersion of the pathways in deionized water, the volume of the liquid increases from a preliminary measurement, *v1*, to a subsequent measurement, *v2*. Upon removal of the conduit, the liquid volume subsequently decreases to a final measurement, *v3*. For each conduit, we calculated the average of three distinct porosity percentages

#### Evaluation of EGCG release in vitro

The in vitro release profile of EGCG from the PCL+EGCG and PCL+EGCG+CNT conduits was evaluated via a UV–visible spectrophotometer (Thermo Fisher, UCA) at a maximum absorption wavelength of 273.5 nm. For this experiment, each conduit was immersed in 20 mL of phosphate-buffered saline (PBS, pH 7.4) and incubated at 37 °C under gentle shaking to simulate physiological conditions. Sampling was performed at predetermined time intervals. At each time point, 5 mL of the release medium was withdrawn and replaced with an equal volume of fresh PBS to maintain sink conditions.

4 $$\begin{gathered} Medication{\text{ }}release{\text{ }}\left( \% \right){\text{ }} \hfill \\= {\text{ }}Amount{\text{ }}of{\text{ }}EGCG{\text{ }}released/Total{\text{ }}EGCG{\text{ }}in{\text{ }}sample \times 100 \hfill \\ \end{gathered} $$

### Cell study

#### Cell seeding on the scaffolds

The fibrous sheets were meticulously cut to fit the size of individual wells in the 48- and 96-well culture plates and subsequently placed inside them. The nanofiber scaffolds were then sterilized via ultraviolet radiation for 40 min. Following sterilization, the scaffolds were washed with PBS at pH 7.4, which contained 1% penicillin/streptomycin and 1% amphotericin B. They were then soaked overnight at 37 °C in DMEM/F12 supplemented with 10% FBS and 1% penicillin/streptomycin. Mesenchymal stem cells (MSCs) were maintained on the nanofiber scaffolds at densities of 6 × 10^4^ and 10^4^ cells in 48-well and 96-well plates, respectively. After 2 h, the samples received additional DMEM/F12 medium containing 10% FBS and were maintained at 37 °C with 5% CO2.

#### Cell adherence and morphology

The formation of proper attachments between engineered polymer scaffolds and cells was examined via SEM after a 4-day culture period. The cell growth medium was subsequently discarded, and the cells were washed with PBS. To facilitate dehydration of the cells adhered to the scaffolds, the samples were initially fixed with Karnovsky’s fixative, which consisted of 2% (w/v) paraformaldehyde and 2.5% (w/v) glutaraldehyde, for 40 min at ambient temperature. A series of ethanol solutions ranging from 30% to 100% were subsequently applied, with each concentration being maintained for 3 min. For SEM analysis, the polymeric scaffolds were coated with gold. Three samples were prepared for each group, each tested in triplicate to ensure reproducibility.

#### Cell proliferation and viability

To investigate cell proliferation and viability, 10^4^ cells were plated in each well of 96-well plates with and without scaffolds, and maintained at 37 °C. The MTT assay was performed at 1-, 3-, and 5- days post-seeding to determine the effects of cell survival and the scaffold on cellular behavior. The scaffolds utilized in this study included PCL, PCL+EGCG, PCL+CNT, and PCL+EGCG+CNT (*n* = 3). After the culture medium was discarded and the wells were washed with PBS, 200 μL of a 0.5 mg/mL MTT solution was added to each well [45]. After a 4 h incubation, the MTT solution was removed, and 100 μL of dimethyl sulfoxide (DMSO) was added to dissolve any formazan crystals that had developed. The mixture was lightly stirred and shaken for 10 min in the dark, and the absorbance at 570 nm was measured via an ELISA reader (Expert 96, Asys Hitch, Austria).

DAPI staining was used to assess cell adhesion at 1-, 3-, and 5- days post-cell implantation onto the scaffolds by selectively staining the MSC nuclei. The staining procedure involved removing the cell culture medium, rinsing the polymeric scaffolds with PBS, and fixing the cells with 4% paraformaldehyde at room temperature for 30 min. An additional PBS wash was performed before the cells were stained with DAPI (10 µg/mL) for 5 min in the dark, ensuring clear visualization of the nuclei. After staining, the DAPI solution was replaced with PBS, which allowed proper imaging. The cell nuclei, attachment, distribution, and viability of the cells on the scaffold were then assessed via fluorescence microscopy (H600L, Optika, Italy) [46, 47].

### In vivo studies

All animal experiments were conducted in accordance with the protocol approved by the Ethical Oversight Committee of Mazandaran University of Medical Sciences (Approval Code: IR.MAZUMS0.4.REC0.1402.18629). The study was designed and reported in compliance with the ARRIVE guidelines to ensure transparency and reproducibility.

#### Operative intervention

A total of 48 adult male Wistar rats, each four months old and weighing between 240 and 280 grams, were obtained from Mazandaran University of Medical Sciences in Sari, Iran. The rats were kept in cages that provided a controlled environment, maintaining a temperature of 23 ± 1 °C, a relative humidity of 55%, and a 12 h light/dark cycle. The animals were treated in accordance with the guidelines set forth by the Animal House of Mazandaran University of Medical Sciences and received standard commercial pellets for nutrition (Behparvar CO), unlimited access to water, and appropriate health care throughout the experimental period.

The sample size (*n* = 8 per group) was determined on the basis of previous studies and power analysis via *G*Power software* (α = 0.05, power = 0.8), which targets functional and histological endpoints. Randomization was performed via a computer-generated sequence to allocate the animals into six groups: (1) control group: no lesion and no treatment (intact sciatic nerve); (2) axotomy group: A 10 mm segment of the sciatic nerve was excised, and both proximal and distal stumps were embedded into the adjacent muscle without any therapeutic intervention; (3) PCL group: Rats with a 10 mm nerve defect underwent PCL-structured NGC treatment; (4) PCL+EGCG group: Rats with a 10 mm nerve defect underwent treatment with PCL+EGCG-structured NGCs; (5) PCL+CNT group: Rats with a 10 mm nerve defect underwent treatment with PCL+CNT-structured NGCs; and (6) PCL+EGCG+CNT group: Rats with a 10 mm nerve defect were treated with PCL+EGCG+CNT-structured NGCs. All animal procedures were conducted in accordance with the ARRIVE and OECD guidelines for ethical handling. The animals were randomly assigned to experimental groups via a computer-generated protocol. For identification, temporary tail color coding was applied via nontoxic markers under light anesthesia prior to randomization. All animals were allowed to recover for at least 24 hours prior to testing to minimize any effects of physiological stress. Investigators responsible for behavioral assessments, histological evaluations, and image analyses were blinded to group allocations during both data collection and interpretation. The individual performing the statistical analysis was also unaware of group identities to minimize analytical bias.

In this study, the right sciatic nerve was designated as the target site for each rat. Surgical intervention began with anesthesia induction via the intraperitoneal injection of ketamine (70 mg/kg, 5%) combined with xylazine (6 mg/kg, 2%), which was administered at a volume of 0.5 mL per rat [48]. To access the sciatic nerve, the skin was surgically opened in the right thigh (Fig. 8A), followed by the establishment of a 10 mm-long injury caused by a cut. The right sciatic nerve was subsequently transected, dividing it into proximal and distal portions. Both sections were kept together via 5–0 nylon sutures, which penetrated 1 mm into a 12 mm-long conduit (Fig. 8C). The conduit was then sutured to connect the injured sciatic nerve, ensuring that both the proximal and distal nerve stumps extended 1 mm into the conduit from each end (Fig. 8D–E).

#### Postsurgical magnetic resonance imaging (MRI) scanning procedure

Following a 12-week recovery period postsurgery, the rats were anesthetized and arranged in a supine orientation for MRI via a 1.5 T scanner (Discovery 750, General Electric). Traditional MRI scanning techniques were applied to acquire T2-weighted images in sagittal, axial, and coronal sections. For the T2-weighted scan, the parameters include a repetition time (TR) of 3000 MS, a matrix dimension of 192 × 192, and a field of view (FOV) of 100 mm. The slice thickness was set at 1 mm without spacing, and the number of excitations (NEX) was recorded as 8.

#### Blood compatibility and hemolysis testing

To evaluate the blood compatibility of the graft materials, hemolysis testing was performed according to ISO standard 10,993–4, which specifies that hemolysis rates should be less than 5% for blood-contacting materials. The NGCs were prepared and immersed in normal saline for 30 min. Fresh blood from the mice was collected and added to the presoaked NGCs, followed by incubation for 1 h. After incubation, the NGCs were extracted, and the mixture was centrifuged at 2000 rpm for 5 min. Photographic documentation was conducted, and the absorbance of the resulting supernatant was quantified at a wavelength of 545 nm. Mouse blood was placed in deionized water to serve as a positive control, whereas normal saline was used as a negative control in the experimental setup.

The hemolysis ratio was calculated via the following equation:

5 $$\begin{gathered} Hemolysis{\text{ }}ratio{\text{ }}\left( \% \right){\text{ }} \hfill \\= {\text{ }}\left( {ODT{\text{ }} - {\text{ }}ODN} \right)/\left( {ODP{\text{ }} - {\text{ }}ODN} \right){\text{ }} \times {\text{ }}100 \hfill \\ \end{gathered} $$

where ODT represents the absorbance value of the test group, ODN denotes the absorbance value of the negative control group, and ODP indicates the absorbance value of the positive control group. This approach provides a quantitative assessment of the blood compatibility of NGCs.

#### Real-timePCR analysis

The quantitative analysis of neurotrophic factors at the site of axotomy was conducted via real-time polymerase chain reaction (real-time PCR) with specific primers for Map2, βIII-tubulin, neurofilament (NF), and p75NTR. Additionally, the expression of the Schwann cell marker gene Schwann100 (S100) was measured (Table 1) [49–51]

**Table 1:** Sequences of primers used for quantitative real-time PCR analysis

Gene	Sequence 5’-3’
**NF**	F: AGCAGGGTCTACAGAGTCAG
	R: GTCCTGTATGTAGCCACTTCC
**MAP2**	F: GCACCTCCACACCTACTACCC
	R: CGGATGATGGCAACTTTCTT
**β tubulin III**	F: TGAGGCCTCCTCTCACAAGT
	R: GTCGGGCCTGAATAGGTGTC
**S100**	F: CAA CCT CCA GAT CCG AGA AA
	R: TCA CAT CAC CAC GCT CTT GT
**P75**	F: CTG AGG GTT TCT CCG GAT TT
	R: TGA AGC CTA TGC TGC ACT TG
**β –actin**	F: GGCCAGACTTTGTTGGATTTG
	R: TGCGCTCATCTTAGGCTTTGT

**F:** Forward; **R:** Reverse; **NF:** Neurofilament; **MAP2:** Microtubule- Associated Protein 2; **S100:** S100 Calcium-Binding Protein; **P75:** p75 Neurotrophin Receptor

Glyceraldehyde-3-phosphate dehydrogenase served as the internal control. Reactions were carried out with SYBR® Premix Ex Taq™ II (Takara Bio, Inc.) on a Rotor-Gene™ 6000 RT‒PCR machine (Corbett Research, Qiagen, Germany). Initial denaturation was performed at 95°C for 15 minutes, followed by 40 cycles of denaturation at 95°C for 5 seconds under primer-specific conditions and extension at 60°C for 20 seconds [52]. Comparative quantitative real-time PCR was performed among the candidate groups via the Relative Expression Software Tool (REST) [50, 51].

#### Histological analysis

This study involved a histological examination of the sciatic nerve, dorsal root ganglia and anterior horn of the spinal cord conducted 12 weeks after the surgical procedures (Fig. 4D–E). The evaluation focused on the following parameters. The total volume at the injury location, nerve fiber density and myelin layer thickness of the sciatic nerve were measured. The overall quantity and numerical concentrations of sensory neurons and glial cells in the dorsal root ganglion (DRG) were assessed. The densities of motor neurons and glial cells in the anterior horn of the spinal cord were quantified.

The tissue was extracted and preserved in 10% formalin for further examination. In accordance with standard tissue processing methods, the samples were embedded in paraffin and sliced with a microtome. Ten divisions per sample were selected for comprehensive analysis. Five divisions were subjected to hematoxylin and eosin (H&E) staining to examine the sciatic nerve volume, nerve fiber density, and histological changes in the DRG and anterior horn of the spinal cord, whereas the other sections were stained with osmium tetroxide and LFB to evaluate the myelin sheath thickness.

#### Stereological analysis

##### Quantitative stereological analysis of sciatic nerve, neuronal, and glial populations in the DRG and Spinal cord

Histological samples were analyzed under 400× magnification, allowing for the enumeration of sciatic nerve fibers and other cellular components within the probe. The numerical densities (NⱴNⱴ) were determined via the following equation:

6 $${N_v}{\text{ }} = {\text{ }}\left( {\Sigma Q/\Sigma p{\text{ }} \times {\text{ }}h{\text{ }} \times {\text{ }}af} \right){\text{ }} \times {\text{ }}t/BA$$

where ΣQ represents the total number of frames counted, h (μm) denotes the height of the dissector, Σp indicates the total number of cells counted within the probe, af (mm^2^) refers to the area of the probe, BA (μm) is the microtome block advance, set at ten μm, and t (μm) signifies the thickness of the section [53].

##### Stereological evaluation of the sciatic nerve, DRG, and spinal cord volume

To assess the total volume of the tissue sections, the Cavalier method was used. This technique requires the overlay of microscopy images of tissue slices arranged on a grid of points, where the points located within the tissue region are counted. The total volume was subsequently determined on the basis of the point calculations via the following formula:

7 $$Vt = \Sigma P \times a/p \times t$$

The total number of counted points (ΣP), the dimensions of each point (approximately mm^2^), and the separation between two consecutive sections (t mm) were recorded [48, 53].

##### Stereological assessment of the thickness of the myelin sheath

The examination of tissue sections was conducted at a magnification of × 100 to estimate the mean myelin sheath thickness. A double line grid, formed by two parallel lines, was systematically overlaid on the sampled areas to determine specific measurement sites. The orthogonal intercept method was used to determine the myelin sheath thickness by measuring the spatial separation from the inner membrane to the outermost layer of the myelin sheath at designated grid intersections. These measurements were termed orthogonal intercepts (oi).

The calculation of the harmonic mean thickness was performed via the specified formula.

8 $$T{\text{ }} = 3.14/4{\text{ }} \times {\text{ }}Ln$$

where Ln represents the intersection points of the axon perimeter with the test lines [53, 54].

#### Gastrocnemius muscle histology and the muscle weight ratio

At 12 weeks post-surgery, the rats were euthanized, and the wet weight loss of the gastrocnemius muscle in the injured group was analyzed against that of the uninjured group via the designated equation for comparison.

9 $$Weight{\text{ }}loss{\text{ }}\left( \% \right){\text{ }} = {\text{ }}(W0 - W1/W0){\text{ }} \times 100$$

The midsections of the muscles were subsequently extracted and placed in a 10% natural formalin solution overnight. The embedded muscles were then sliced into transverse sections of 5 µm thickness via a microtome. These samples were subjected to H&E staining and analyzed via light microscopy.

#### Immunohistochemical analysis of Schwann cells and axonal regeneration

To evaluate the presence of Schwann cells in the sciatic nerve, antibody staining for the S100β antibody was performed 12 weeks after the surgical procedure. For this study, five sections were selected from each rat at uniform spaces. After deparaffinization, all segments were treated with goat serum to decrease nonspecific binding. The sections were incubated overnight at 4 °C with the S100β antibody (1:500 in PBS, Santa Cruz Biotechnology sc-393919). After incubation, the sections were washed with PBS and treated with a secondary antibody. Positive responses were visualized via the use of diaminobenzidine tetrahydrochloride. A density measurement technique was employed to quantify the density of successful reactions, ensuring accurate assessment of Schwann cells.

The process of axon outgrowth is critically influenced by various factors. In this investigation, an NF-200 antibody was utilized to specifically target the 200-kDa neurofilament protein, enabling precise visualization of axonal regeneration within the conduits. The methodology consisted of several key steps. The tissue blocks were placed on ice and sectioned to a thickness of 4–6 μm. Sections were stained with both NF-200 antibodies (Duccio-Artiuo, Abcam, USA). A secondary antibody (Envision Templas Dakumus) was subsequently applied to the tissue samples. The tissues were stained with diaminobenzidine (DAB), after which counterstaining was performed with hematoxylin.

#### Assessment of proinflammatory cytokines

An enzyme-linked immunosorbent assay (ELISA) was used to determine the levels of proinflammatory cytokines, such as IL-1β, TNF-α, and IFN-γ. The procedure is detailed as follows:

A tissue sample weighing 100 mg was processed in lysis buffer containing the following components: 1 mM benzylsulfonyl fluoride, 1% Triton X-100, 2 mM EDTA, 2.5 mM tetrasodium phosphate, 20 mM Tris, and 0.5 μg/mL N-acetyl-L-leucyl-L-leucyl-L-arginine. The mixture was centrifuged at 15,000 rpm for 15 min to obtain the cell pellet. The quantification of cytokine levels was performed via ELISA. [54]

### Functional assessments

#### The sciatic function index and tibial function index

##### Evaluation of motor function recovery

Regained motor ability was measured via the sciatic function index (SFI) and tibial functional index (TFI), in accordance with previous studies [11]. Evaluations were conducted at 0, 4, 8, and 12 weeks after the surgical procedure. In summary, the treated rats were permitted to navigate a wooden corridor (100 cm × 10 cm × 10 cm) within a darkened environment, where black and white papers were strategically placed at the end and on the floor, respectively. The posterior limbs of the rats were dipped in black ink to capture their footprints on the paper during movement. The calculation of the SFI and TFI was performed via the following equation:

10 $$\begin{gathered} Sciatic{\text{ }}function{\text{ }}index{\text{ }} \hfill \\= {\text{ }} - 38.3{\text{ }}(\left[ {EPL - {\text{ }}NPL} \right]/NPL + 109.5{\text{ }}\left( {\left[ {ETS - {\text{ }}NTS} \right]/NTS} \right){\text{ }} \hfill \\+ 13.3{\text{ }}\left( {\left[ {EIT - {\text{ }}NIT} \right]/NIT} \right){\text{ }} - {\text{ }}8.8. \hfill \\ \end{gathered} $$

11 $$\begin{gathered} Tibial{\text{ }}functional{\text{ }}index \hfill \\= {\text{ }} - {\text{ }}37.2{\text{ }}\left( {\left[ {EPL - NPL} \right]/NPL} \right){\text{ }} \hfill \\+ {\text{ }}104.4{\text{ }}\left( {\left[ {ETS - NTS} \right]/NTS} \right){\text{ }} \hfill \\+ {\text{ }}45.6{\text{ }}\left( {\left[ {EIT - NIT} \right]/NIT} \right){\text{ }} - {\text{ }}8.8. \hfill \\ \end{gathered} $$

EPL: experimental paw length, NPL: normal paw length, ETS: experimental toe spread, NTS: normal toe spread, EIT: experimental intermediate toe spread, NIT: normal intermediate toe spread. This approach facilitates the longitudinal evaluation of motor function recovery subsequent to surgical intervention [48, 55].

##### Hot plate latency analysis for sensory function recovery

A thorough evaluation of sensory function recovery was performed in the rats 12 weeks after surgery via the hot plate latency (HPL) test. For this procedure, damaged limbs were placed on a hot plate at 56 °C. The latency to a defined behavioral response, such as paw licking or jumping, was meticulously recorded. To ensure ethical compliance and prevent any undue distress, a cutoff response time was strictly set at 10 seconds. This quantitative measure provides an index of nociceptive recovery in treated rats.

#### Paw withdrawal thresholds (PWTs)

All assessments of pain behavior were conducted by a skilled investigator who remained unaware of the experimental conditions. Inflammatory pain sensitivity is characterized by a decrease in paw withdrawal thresholds (PWTs) in response to nonnoxious mechanical input. PWTs were measured via an automated von Frey-type apparatus (Dynamic Plantar Aesthesiometer 37,450, UGO Basile, Italy), according to previous studies. Before the experimental procedures commenced, the rats participated in two training sessions to acclimate them to the testing environment. They were positioned on a metal mesh surface and allowed a 30 min adaptation period. Through the base of the plastic test chamber, a mechanical stimulus was applied to the plantar area of the right hind paw. A steel rod with a diameter of 0.5 mm was used to apply increasing force on the hind paw, ranging from 0 to 50 g over a duration of 20 sec. The mechanical stimulus was automatically stopped when the hind paw of the rat withdrew. The force at which the withdrawal occurred was recorded to the nearest 0.1 g, ensuring precise measurement of the paw withdrawal threshold.

#### Electromyography latency test for muscle reaction evaluation

An electromyography (EMG) latency assessment was conducted to analyze the muscular response to nerve stimulation. The procedure comprises several key steps: The subjects are anesthetized to reduce any potential discomfort throughout the process. Surgery was performed to access the sciatic nerve, facilitating accurate electrode placement. The stimulating electrode was affixed to the proximal segment of the nerve adjacent to the gluteal muscles, and the receiving electrode was positioned on the gastrocnemius muscle. After nerve stimulation, the rate of muscle response was monitored. The EMG latency, defined as the interval between nerve stimulation and subsequent muscle response, was quantified in milliseconds. This assessment yielded significant information regarding the functional recovery of nerve and muscle interactions following treatment. Nerve conduction velocity (NCV) was assessed before the operation and after 12 weeks in the control, axotomy, and control groups, in accordance with a prior protocol. While anesthetized, the sciatic nerve receives single electrical pulses lasting 200 μs, and supramaximal activation is optimized for peak amplitude output [56].

### Statistical analysis

Data processing, statistical analyses, and visualization were performed via GraphPad Prism software (version 10; GraphPad Software, San Diego, CA, USA). The data are presented as the means ± deviations (SDs). Comparisons between two groups were performed via unpaired t-tests. For multiple group comparisons, two-way ANOVA followed by Tukey’s post hoc test was applied, as appropriate. The 95% confidence intervals (CIs) were calculated to estimate the precision of the mean differences. A *p* value of less than 0.05 was considered statistically significant. (*p < 0.05; **p < 0.01; *** *p* < 0.001; **** *p* < 0.0001).

## Results

### Analysis of scaffold properties

#### SEM analysis

**Fig. 3:**
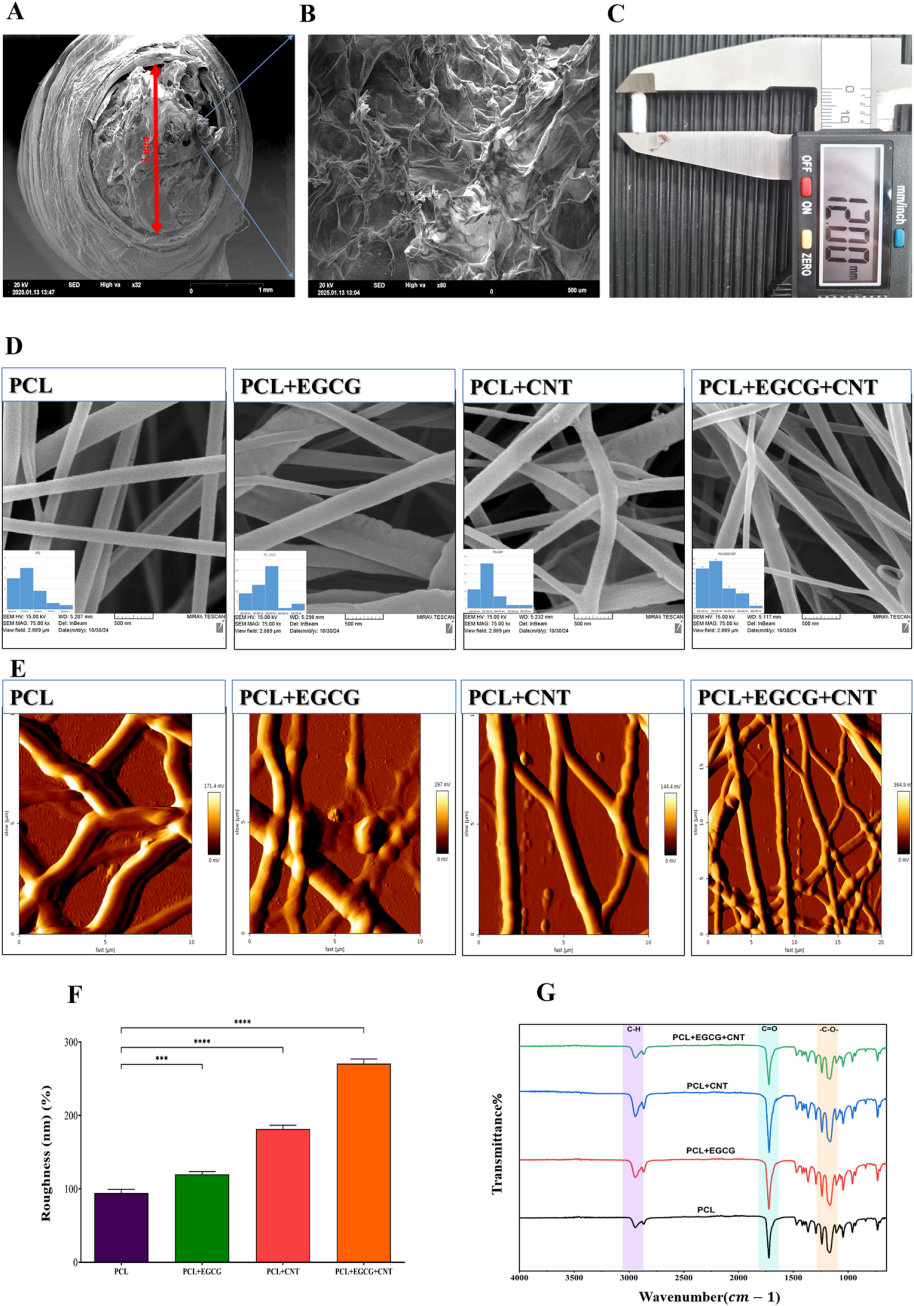
Morphological, topographical, and chemical characterization of the collagen-filled electrospun scaffolds. (**A**) SEM image showing the inner architecture of the fabricated conduit filled with a collagen hydrogel. (**B**) Magnified SEM image of collagen fibers coating the inner lumen of the conduit. (**C**) macroscopic image of the collagen-filled conduit prior to implantation. (**D**) SEM micrographs and corresponding diameter histograms of nanofibers in four scaffold formulations: PCL, PCL+EGCG, PCL+CNT, and PCL+EGCG+CNT. Compared with PCL alone, CNT incorporation significantly reduced the fiber diameter (*p* < 0.001, PCL vs PCL+CNT and PCL vs PCL+EGCG+CNT; mean ± sd, *n* = 3). (**E**) AFM images of scaffold surfaces showing increased roughness in the CNT- and EGCG-containing groups. (**F**) quantitative roughness analysis revealed significantly greater surface roughness in the PCL+EGCG+CNT group than in the PCL group (****p < 0.0001, mean ± sd, *n* = 3). (**G**) FTIR-ATR spectra confirming the chemical integration of EGCG and carboxyl-functionalized CNTs within the PCL matrix. The characteristic peaks at 1722, 1441, and 1364 cm^− 1^ indicate successful composite formation. All scale bars: 1 mm (**A**), 500 µm (**B**), 500 nm (**E**) and 10 µm (**E**)

SEM images revealed that the average diameter of the different scaffolds was as follows: the inner diameter was 3.1 ± 0.3 mm, and the outer diameter was 3.3 ± 1.2 mm (mean ± SD, *n* = 3; Fig. 3A), the dimensions of which are consistent with those of the native rat sciatic nerve (Fig. 3A). Quantitative analysis of nanofiber diameters revealed significant differences among the four scaffold groups (Fig. 3D). The average diameters were as follows: PCL (235 ± 1.45 nm), PCL+EGCG (203 ± 2.21 nm), PCL+CNT (180 ± 1.31 nm), and PCL+EGCG+CNT (157 ± 2.15 nm), all reported as the means ± SD (*n* = 3). Two-way ANOVA followed by Dunnett’s post hoc test confirmed that, compared with PCL alone, CNT incorporation significantly reduced the fiber diameter (*p* < 0.001, PCL vs PCL+CNT and PCL vs PCL+EGCG+CNT). The decrease in diameter is attributed to increased solution conductivity and electrostatic stretching during electrospinning. This morphological refinement, along with increased surface area, may increase EGCG release kinetics and scaffold–cell interactions, contributing to the improved regenerative outcomes observed in subsequent analyses.

#### Atomic force microscopy (AFM)

The morphology and dimensions of the nanofiber assemblies were verified via AFM, confirming their size and diameter with high resolution. The analysis demonstrated that the PCL fibers retained consistent diameter relationships, with measured diameters of 100, 150, and 200 nm (Fig. 3E). The roughness of PCL fibers incorporated with EGCG and CNTs was significantly greater than that of pure PCL fibers. The PCL+EGCG+CNT fibers have a roughness of 300 nm, whereas the other samples have a roughness of 100 to 210 nm (Fig. 3F).

#### FTIR‒ATR spectroscopy of the scaffolds

FTIR-ATR analysis of the PCL+EGCG+CNT mixture revealed key chemical interactions and structural characteristics. The notable peak at 1722 cm^− 1^ indicates the presence of the O = C-O functional group in the PCL scaffold [57]. The C-O-C stretching band at 1177 cm^− 1^ confirms the polymeric nature of PCL [58], whereas the 2943 cm^− 1^ band is linked to C-H stretching in the CH₃ group [57]. Additional peaks at 1364 and 1441 cm^− 1^ correspond to CNTs functionalized with carboxylic groups (COOH) [59], as noted in the literature. The 1720 cm^− 1^ peak, attributed to carboxylic group stretching, suggested that nitric acid oxidation facilitated the attachment of these groups to the CNTs [60]. These spectral features highlight the successful functionalization and interaction among the components of the PCL+EGCG+CNT mixture, demonstrating the efficacy of the material modification process (Fig. 3G).

#### Evaluation of four-point probe measurements

On the basis of evaluations of the electrical resistance and conductivity properties of the conduits, we concluded that CNTs provide a satisfactory level of electrical conductivity to the fabricated conduits. The difference in conductivity between the PCL+CNT and PCL+EGCG+CNT groups was not statistically significant (Table 2).

**Table 2:** Electrical resistivity and conductivity of CNT-containing electrospun scaffolds. Quantitative comparison of the electrical properties of the PCL+CNT and PCL+CNT+EGCG conduits. Measurements were performed at room temperature via a four-point probe method. The data are presented as the means ± SDs (*n* = 3). Although the addition of EGCG slightly increased the resistivity and reduced the conductivity, the difference between the two groups was not statistically significant (*p* > 0.05). CNT incorporation provided stable and satisfactory conductivity across both formulations

Polymer	Electrical resistivity y(ρ) Ω.cm	Electrical conductivity (σ) S.cm−1
**PCL + CNT**	0.291 ± 0.052	3.43 ± 0.64
**PCL + CNT + EGCG**	0.384 ± 0.073	2.60 ± 0.50

#### Rate of degradation and pH variability

**Fig. 4:**
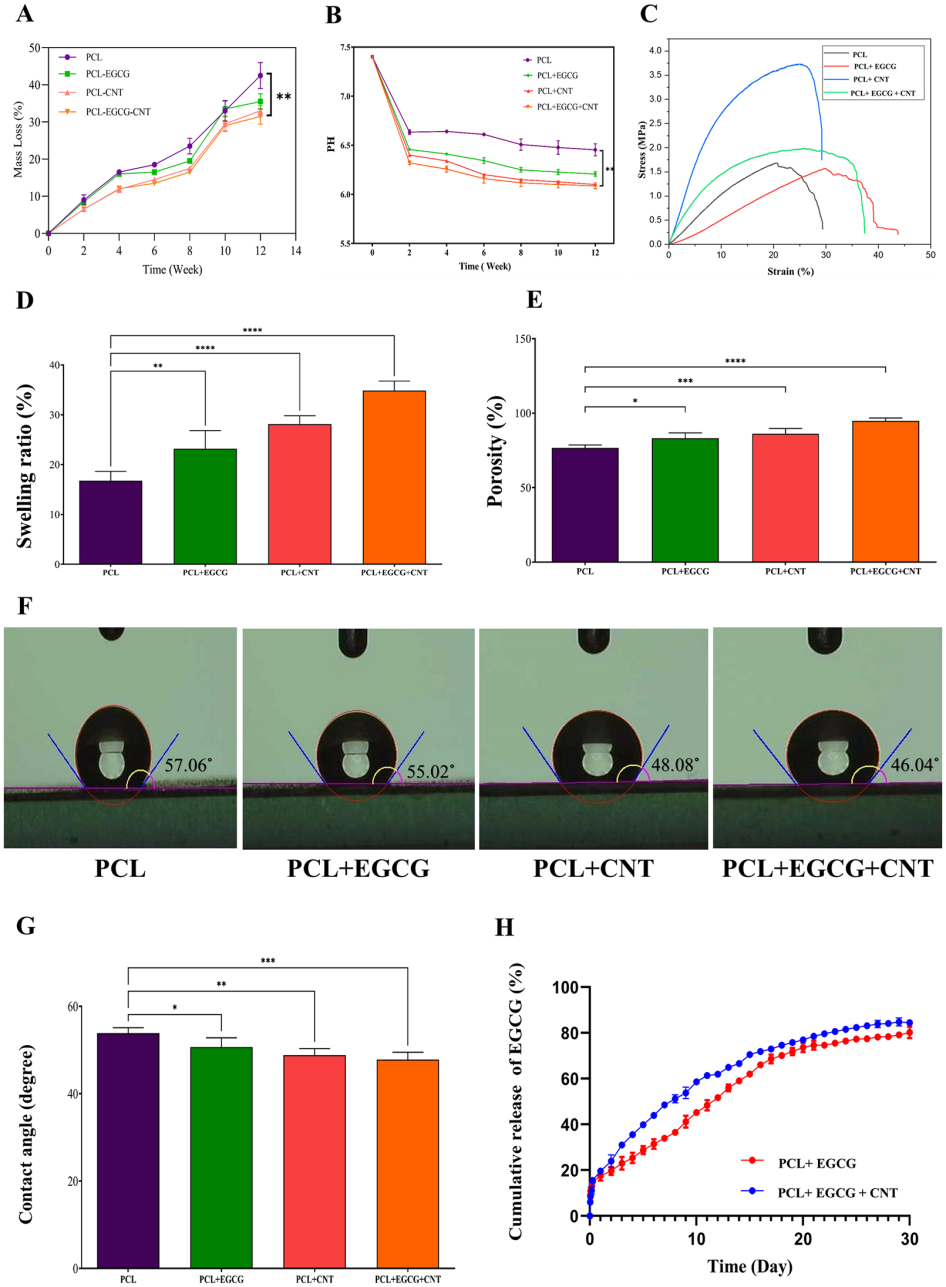
Physicochemical and mechanical characterization of PCL-based nerve conduits. (**A**) rates of degradation and (**B**) pH variation of PCL, PCL+EGCG, PCL+CNT, and PCL+EGCG+CNT conduits during 12 weeks of incubation in PBS (pH 7.4) at 37 °C. All the groups exhibited a gradual and significant change in the degradation rate over time (*p* < 0.05). (**C**) tensile properties of the conduits measured under a 10 N tensile load and an extension rate of 1 mm/min at 37 °C, showing that CNT incorporation improved the mechanical strength while maintaining flexibility (**D**) the swelling ratios of the PCL, PCL+EGCG, PCL+CNT, and PCL+EGCG+CNT scaffolds after 48 h of incubation at 37 °C with PBS (pH 7.4). **(E**) porosity ratio and (**F**) changes in the contact angle of the nanofibrous mats, where the addition of CNTs increased the hydrophilicity and surface wettability of the constructs. (**G**) the addition of MWCNTs increased the hydrophilicity of the PCL nanofibers. The data are reported as the means ± SDs, *n* = 3 (*p < 0.05, **p < 0.01, ***p < 0.001). (**H**) EGCG release from the PCL+EGCG and PCL+EGCG+CNT samples. All the samples had equal amounts of initial encapsulated EGCG. Significant differences are shown as *p < 0.05, **p < 0.01, and ***p < 0.001

The in vitro degradation of the conduits was evaluated by observing the pH and mass loss variations over a 12-week period. The results are depicted in Figure, which illustrates the temporal changes in both weight and pH. The conduits immersed in PBS presented average percentages of weight loss at each time interval (Fig. 4A).

The degradation rates across all groups changed significantly throughout the 12-week period (*p* < 0.05). The incorporation of CNTs within the conduit walls significantly influences their physical and mechanical characteristics. These investigations confirm the stability of the CNTs within the conduit structure. The pH changes in the PCL, PCL+EGCG, PCL+CNT, and PCL+EGCG+CNT scaffolds are shown. A decrease in pH was observed at week 12, with values of 6.56, 6.35, 6.15, and 6.08, respectively. (*p* > 0.05) (Fig. 4B).

#### Tensile test analysis

The tensile strength analysis provided further insight into the mechanical properties of the developed NGCs. Compared with the PCL scaffold (3.39 MPa, *p* < 0.05), the PCL+EGCG+CNT (9.74 MPa) scaffold had significantly greater tensile strength. Similarly, the PCL+EGCG (5.24 MPa) conduits demonstrated increased tensile strength, although this change was not statistically significant compared with that of the PCL scaffold.

(3.39 MPa, *p* > 0.05)

The incorporation of CNTs into the PCL (14.63 MPa, *p* < 0.01) conduits significantly enhanced the tensile strength of the NGCs. This substantial improvement highlights the essential function of CNTs in reinforcing the structural integrity of conduits. These findings suggest that adding CNTs to the PCL matrix not only increases the mechanical robustness of conduits but also potentially enhances their applicability in nerve regeneration therapies by providing stronger and more durable scaffolds (Fig. 4C and Table 3).

**Table 3:** Young’s modulus and ultimate tensile strength (uts) of PCL, PCL + EGCG, PCL + CNT, and PCL + EGCG + CNT. Tensile property measurements were performed under a 10 n tensile load and an extension rate of 1 mm/min at 37 °C. uts: ultimate tensile strength, Ɛb: elongation at break. The data are presented as the means ± SDs, *n* = 3

Group	Young ҆s modulus (MPa)	UTS (MPa)	Ɛb **(%)**
PCL	3.39 ± 4.6	0.65 ± 0.13	14.38 ± 2
PCL+EGCG	5.24 ± 1.9	0.48 ± 0.9	29.33 ± 12
PCL+CNT	14.63 ± 1.59	0.35 ± 1.8	37.12 ± 6
PCL+EGCG+CNT	9.74 ± 2.4	0.25 ± 3.6	43.72 ± 2

#### Swelling ratio of conduits

The extent of swelling in polymer-based scaffolds is influenced by several critical factors, including polymer interactions, surface characteristics, and crosslinking density. Together, these elements determine the overall swelling capacity of the scaffold, which is vital for its efficacy in various biomedical applications, including tissue engineering [36, 37, 44]. The figure presents the water absorption percentages in the polymeric conduits after two days of immersion in PBS. Among the tested groups, the PCL and PCL + EGCG scaffolds presented relatively low swelling ratios, reflecting their hydrophobic nature and limited water uptake. In contrast, the PCL + CNT group presented a significantly greater swelling percentage than the PCL alone group did (*p* < 0.05, PCL vs PCL+CNT), likely due to the increased surface area and porosity introduced by the CNTs. Furthermore, the PCL + EGCG+CNT scaffold resulted the greatest water absorption among all the groups, significantly exceeding both the PCL+EGCG and PCL+CNT groups (*p* < 0.01, PCL+EGCG+CNT vs PCL+EGCG; *p* < 0.05, PCL+EGCG+CNT vs PCL+CNT). These findings suggest a synergistic effect of EGCG and CNTs in enhancing scaffold hydrophilicity and swelling behavior (Fig. 4D).

#### Porosity assessment

The developed conduits exhibit a porosity level exceeding 70%, enhancing their compatibility for tissue engineering applications [11]. Among the tested formulations, the PCL + EGCG + CNT scaffold demonstrated the highest porosity at 93.5%, which was significantly greater than that of the PCL (71.2%), PCL+EGCG (76.8%), and PCL+CNT (82.4%) conduits (*p* < 0.01, PCL+EGCG+CNT vs all other groups; Fig. 4E). This enhanced porosity is likely due to the synergistic effect of EGCG and CNTs in modifying the fiber architecture and increasing the interfiber spacing.

#### Assessment of contact angle values

The surface properties of fibrous scaffolds are crucial for influencing initial cell adhesion, multiplication, and migration. This phenomenon was assessed through contact angle measurements using deionized water. The water contact angles of the various nanofibrous mats were recorded as follows: PCL (57.06 ± 1.4°), PCL+EGCG (55.02 ± 1.8°), PCL+CNT (48.02 ± 0.8°), and PCL+EGCG+CNT (46.04 ± 2.8°). Compared with the PCL (*p* < 0.01), PCL+EGCG (*p* < 0.05), and PCL+CNT (*p* < 0.05) groups, the PCL+EGCG+CNT group presented a significantly lower contact angle, indicating enhanced surface hydrophilicity due to the synergistic effect of EGCG and functionalized CNTs (Fig. 4F–G).

#### Controlled release of EGCG from conduits

The evaluation of EGCG release from the conduit demonstrated a regulated release mechanism. In the cases of the PCL+EGCG and PCL+EGCG+CNT conduits, there was no abrupt drug release within the initial 24-hour period, as illustrated in the figure (Fig. 4H). The peak release occurred on day 30, which can be attributed to the superior integration achieved in the conduit fabrication. These findings indicate that the PCL+EGCG+CNT conduits facilitated the sustained release of EGCG, ensuring consistent and prolonged delivery for enhanced regeneration in vivo. This controlled release mechanism is essential for maintaining therapeutic EGCG levels over an extended period, thereby optimizing the efficacy of conduits in supporting nerve regeneration.

### Cell study

#### The morphology and interactions of mesenchymal stem cells

**Fig. 5:**
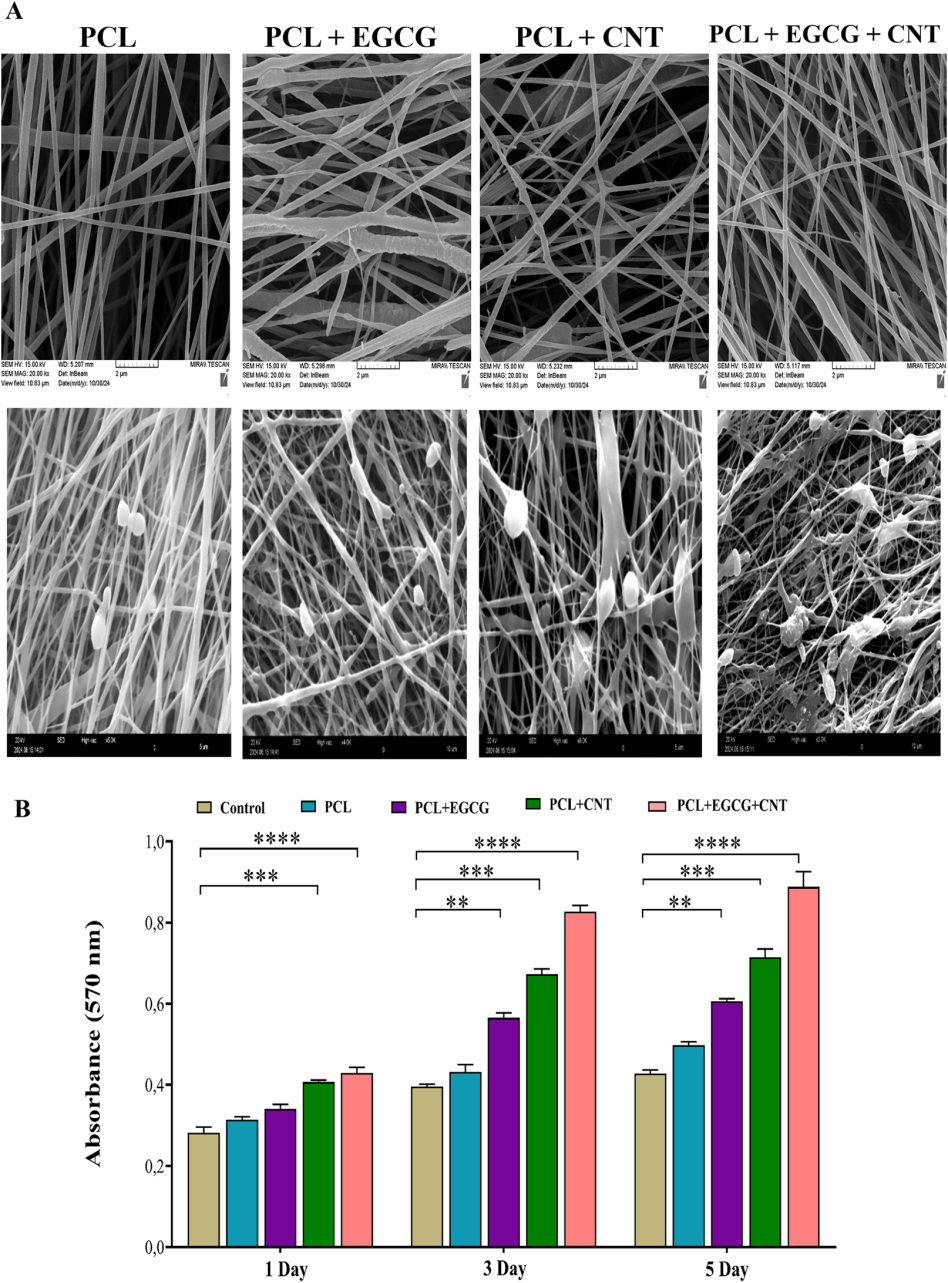
(**A**) SEM images of MSCs cultured on PCL, PCL+EGCG, PCL+CNT, and PCL+EGCG+CNT nanofibrous scaffolds after 4 days. Scaffolds without cells and scaffolds with cells. (**B**) MTT assay. The viability of MSCs seeded on tissue culture plates (TCPs) and different nanofbrous scaffolds after 1, 3, and 5 days. The data are presented as the means ± SDs (*n* = 3). Significant differences are shown as *p < 0.05, **p < 0.01, and ***p < 0.001

After a 4-day incubation period, the scaffolds were examined via SEM. SEM images revealed that the MSCs successfully attached to and proliferated throughout all the scaffold types (Fig. 4A). These visual representations highlight the dynamic interactions between the scaffolds and the cells, illustrating the effective adhesion of the cultured cells to the scaffold surfaces. Furthermore, the porous microarchitecture of the scaffolds facilitates the delivery of sufficient oxygen and nutrients to both the embedded constructs and the cultured cells. This characteristic is essential for maintaining cell viability and fostering effective tissue regeneration. These findings indicate that the use of CNTs improved cell adhesion. EGCG and CNTs have a synergistic effect on cell adhesion and better connections. Ultimately, the PCL+EGCG+CNT nanofibrous scaffolds resulted in better cell interactions, as shown in Fig. 5A.

#### MTT assay and fluorescence imaging results

The quantitative evaluation of cellular viability following adhesion and development on the nanocomposite mats was conducted via the MTT assay. The study included various samples, including tissue culture plates (TCPs), PCL, PCL+EGCG, PCL+CNT, and PCL+EGCG+CNT nanofibrous scaffolds. This evaluation determines the concentration of generated purple formazan crystals, which is directly related to the number of living cells. To facilitate the dissolution of the formazan product and achieve the characteristic purple color, DMSO was used as the solvent [61]. Data were collected on days 1, 3, and 5 postseeding. All the scaffold groups demonstrated favorable cytocompatibility over time, with increasing absorbance values indicating progressive cell proliferation. Notably, the PCL+EGCG+CNT group presented significantly greater cell viability than the TCP group did on all the assessed days (*p* < 0.05 on day 1, *p* < 0.01 on day 3, and *p* < 0.001 on day 5; PCL+EGCG+CNT vs TCP). Additionally, this group showed statistically significant improvements over the PCL and PCL+EGCG groups on days 3 and 5 (*p* < 0.05), suggesting a synergistic effect of EGCG and CNTs in enhancing scaffold bioactivity (Fig. 5B).

**Fig. 6:**
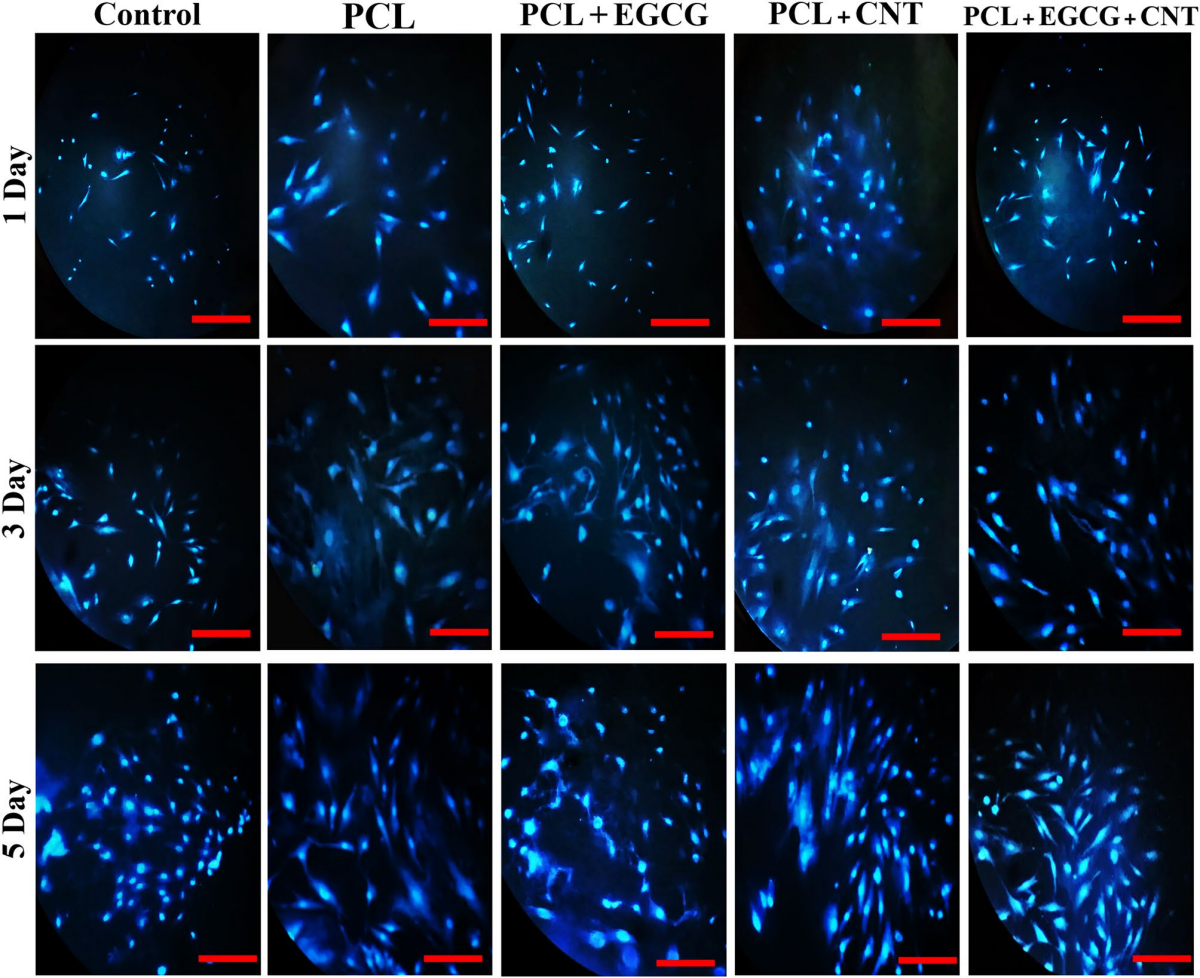
Fluorescence microscopy images of mesenchymal stem cells (MSCs) stained with DAPI and cultured on tissue culture plates (TCPs) and nanofibrous scaffolds (PCL, PCL+EGCG, PCL+CNT, and PCL+EGCG+CNT) after 1, 3, and 5 days. DAPI staining highlights cell nuclei in blue, enabling visualization of cell attachment and distribution across different substrates. Scale bars = 100 µm

The PCL+EGCG+CNT groups were significantly different from the other groups at all three time points. Fluorescence imaging was utilized to visualize cells stained with DAPI, with a focus on those cultured in various environments. Under fluorescence microscopy, the nuclei appeared blue, allowing for a clear comparison of cell attachment across different scaffolds. The results revealed a markedly greater number of adherent cells on the PCL+CNT and PCL+EGCG+CNT scaffolds than on the PCL and PCL+EGCG scaffolds. Among all the groups, the PCL+EGCG+CNT scaffold exhibited the most extensive cell coverage and nuclear density, indicating enhanced cellular affinity. Quantitative analysis confirmed statistically significant differences in cell attachment between the PCL+EGCG+CNT group and all other groups at each assessed time point (*p* < 0.05, PCL+EGCG+CNT vs PCL, PCL+EGCG, and PCL+CNT) (Fig. 6).

### In vivo study

#### MRI evaluation of sciatic nerve pathology

**Fig. 7:**
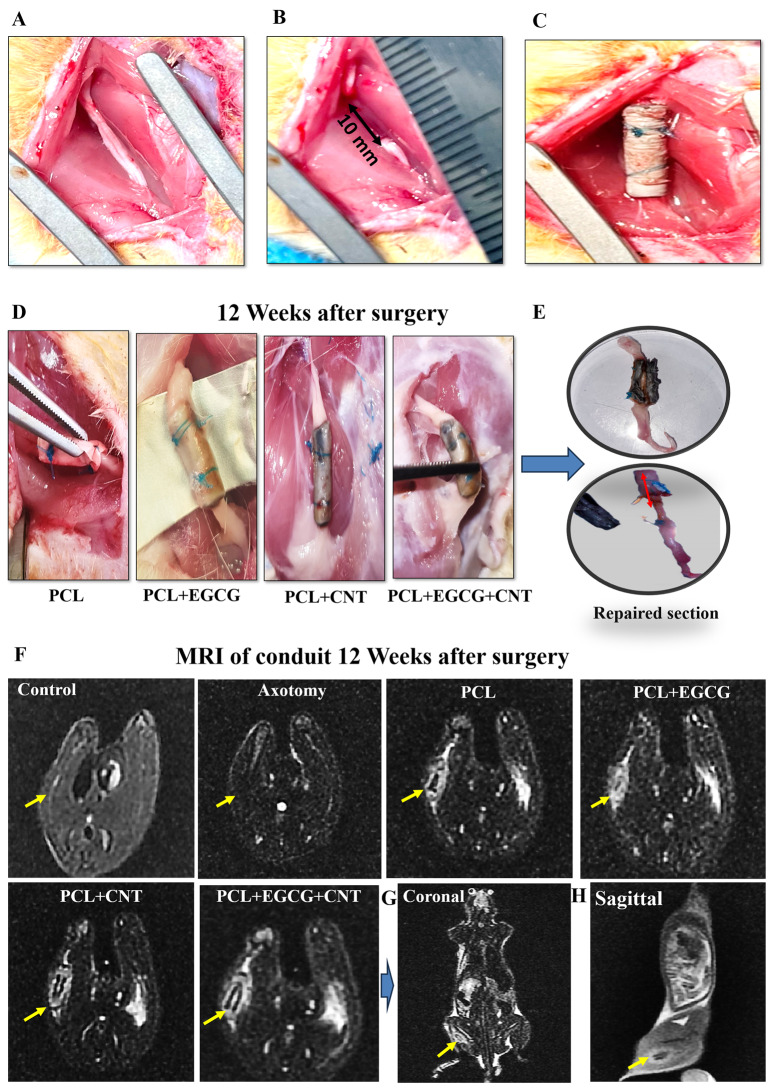
(**A**) The sciatic nerve of the rat was obtained via a skin incision, and (**B**) a 10 mm-long sciatic nerve was cut. (**C**) The prepared conduit was sutured at the site of injury to prevent sciatic nerve injury. (**D**) a 12-week recovery period after surgery. (**E**) repaired section. (**F**) MR image of the operated and normal sciatic nerve in axial sections. Following a 12-week recovery period after surgery, the rats were anesthetized and arranged in a supine orientation for imaging via a 1.5 T MRI scanner (discovery 750, General electric). (**G**-**H**) MRI scanning techniques were used to acquire T2-weighted images of sagittal and coronal sections from the PCL+EGCG+CNT group

MRI was used to examine the condition of the injured sciatic nerve 12 weeks after surgical intervention. Imaging revealed several notable findings, including the presence of irregular cavities and fibrous scar tissue at the surgical site, which are likely indicative of tissue loss resulting from the procedure. Additionally, both the nerve structure and the surrounding extracellular matrix were examined, offering a detailed perspective on the ongoing repair mechanisms. The MRI results also revealed a reduction in the size of the cavity at the site of contusion over the twelve-week period, suggesting that the infiltration and proliferation of nerve fibers contributed to this decrease. These observations underscore the progress in the restoration of the sciatic nerve (Fig. 7F). The MR images revealed that neural recovery was significantly enhanced in the PCL+EGCG+CNT-treated subjects. Consequently, both coronal and sagittal views were examined, and nerve reconstruction was confirmed (Fig. 7G–H).

#### Gastrocnemius muscle wet weight and histology

**Fig. 8:**
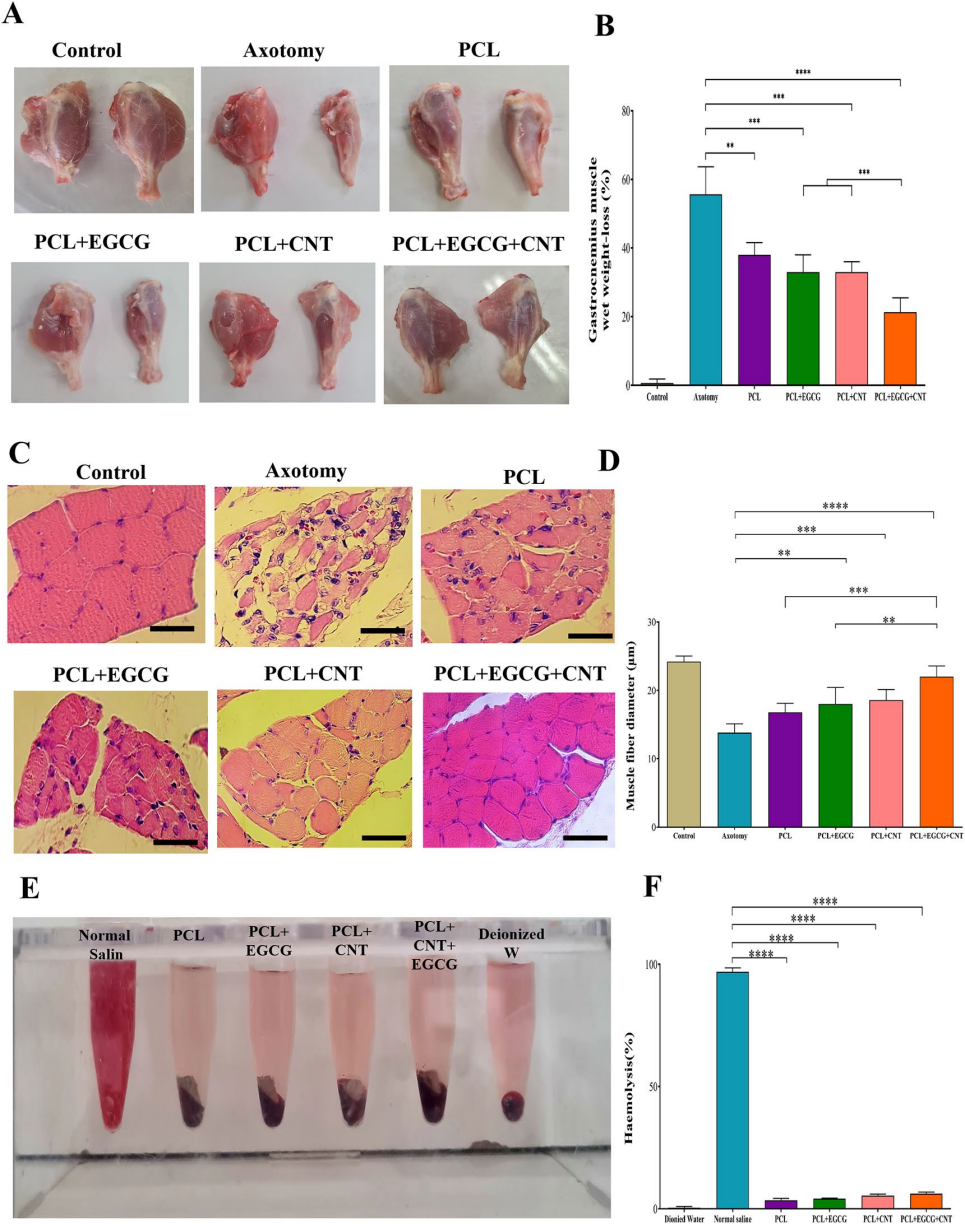
(**A**) Representative images of transverse gastrocnemius muscle sections following H&E staining at 12 weeks after surgery (scale bars = 100 µm). (**B**) The mean diameters of muscle fibers. (**C**) Images of the gastrocnemius muscles related to the normal sciatic nerve to the injured sciatic nerve. (**D**) Wet muscle weight ratio (%) and the impact of PCL loaded with EGCG and CNTs on muscle weight. This study measured the weight of the gastrocnemius muscle on the injured side to assess the impact of sciatic nerve transection and the effectiveness of PCL loaded with EGCG and CNTs in preventing damage. (**E**) Visual assessment of hemolysis in blood samples incubated with different scaffold materials (PCL, PCL+EGCG, PCL+CNT, PCL+EGCG+CNT), deionized water (negative control), and normal saline (positive control). (**F**) Quantitative hemolysis ratios (%) calculated from absorbance measurements after 30 minutes of coincubation, confirming the hemocompatibility of all scaffold groups. The data are presented as the means ± SDs (*n* = 3). The data are presented as the means ± SDs (*n* = 3). Statistical significance is indicated as *p < 0.05; **p < 0.01, ***p < 0.001, ****p < 0.0001

To evaluate the impact of sciatic nerve transection and the therapeutic potential of PCL-based conduits loaded with EGCG and CNTs, the mass of the gastrocnemius muscle on the injured limb was quantitatively assessed. Muscle weights, including axotomy (no treatment), PCL, PCL+EGCG, PCL+CNT, and PCL+EGCG+CNT, weights were compared across groups. Compared with the axotomy group, all the scaffold groups presented significantly greater muscle mass (*p* < 0.01 for PCL, *p* < 0.001 for PCL + EGCG and PCL + CNT, and *p* < 0.0001 for PCL+EGCG+CNT), indicating reduced muscle atrophy and improved functional recovery (Fig. 8A–B). Furthermore, when the scaffold groups were compared, the PCL+EGCG+CNT group presented significantly greater muscle mass than the PCL (*p* < 0.01), PCL+EGCG (*p* < 0.05), and PCL+CNT (*p* < 0.05) groups did, suggesting a synergistic effect of EGCG and CNTs in mitigating denervation-induced muscle loss. Histological analysis of gastrocnemius muscle fibers was performed via hematoxylin and eosin (H&E) staining 12 weeks after conduit implantation. In the axotomy group, muscle fibers appeared to shrink, with increased infiltration of inflammatory cells. In contrast, the scaffold-treated groups presented improved fiber morphology and reduced inflammation. Notably, the PCL+EGCG+CNT group presented more peripheral nuclei and well-organized muscle fibers with near-normal architecture than the other groups did (Fig. 8C–D).

#### Hemolysis ratio assays

Hemolysis ratios were evaluated in the experimental groups, which included PCL, PCL+EGCG, PCL+CNT, and PCL+EGCG+CNT. Deionized water served as the baseline control, and normal saline served as the positive control. Visual evaluations of the PCL, PCL+EGCG, PCL+CNT, and PCL+EGCG+CNT solutions after 30 min of coculture with blood revealed a bright yellow color, whereas the normal saline solution presented a distinct red color, which occurred as a result of hemolysis. The absorbance measurements of the supernatants confirmed the hemolysis ratios: PCL, 1.61%; PCL+EGCG, 2.78%; PCL+CNT, 3.92%; PCL+EGCG+CNT, 4.27%; and normal saline, 99%. All the tested conduits exhibited high biocompatibility, and with a low hemolysis ratio, they have high potential for repair applications (Fig. 8E–F).

#### Histological evaluation of the impact of PCLs containing EGCG and CNTs on the progression of sciatic nerve injury

**Fig. 9:**
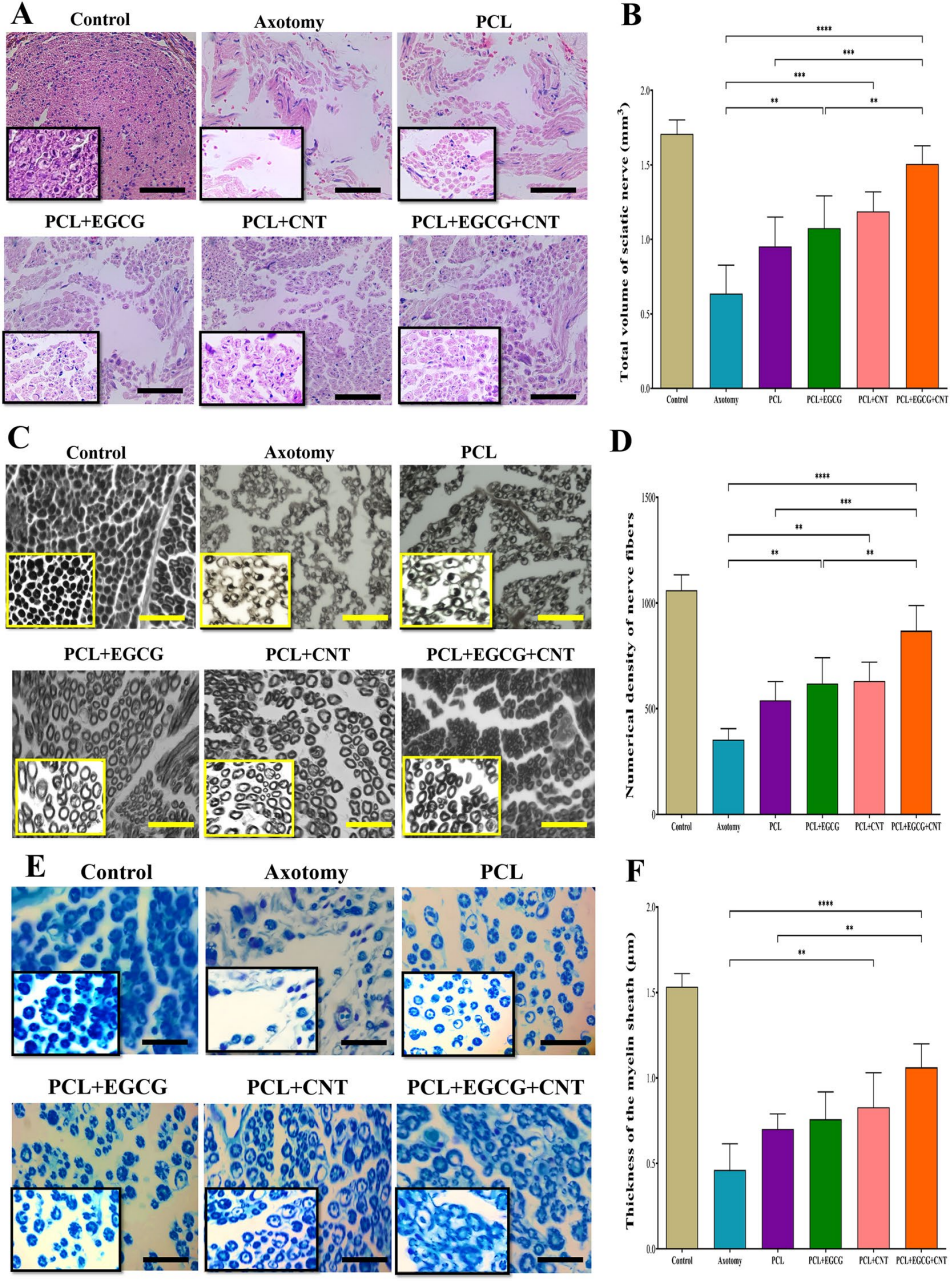
Histological evaluation of sciatic nerve regeneration following conduit implantation. (**A**) Representative transverse sections of regenerated sciatic nerves stained with hematoxylin and eosin (**H**&**E**) at 12 weeks post-surgery (scale bar = 50 µm). (**B**) quantitative analysis of total nerve volume determined via Cavalieri’s method. (**C**, **E**) cross-sections of sciatic nerves stained with osmium tetroxide and Luxol Fast blue (LFB), highlighting myelin sheath integrity and distribution (scale bar = 50 µm). (**D**, **F**) quantification of myelin sheath thickness across the experimental groups. The data are presented as the means ± SDs (*n* = 3). Statistical significance is indicated as *p < 0.05; **p < 0.01, ***p < 0.001, ****p < 0.0001

Histological evaluation of sciatic nerve regeneration was performed via hematoxylin and eosin (H&E) staining at 12 weeks post-surgery. Figure 9A shows representative transverse sections of the injured site across the experimental groups. Compared with those in the axotomy group, the overall nerve volume in the PCL (*p* < 0.05), PCL+EGCG (*p* < 0.01), PCL+CNT (*p* < 0.001), and PCL+EGCG+CNT (*p* < 0.0001) groups was significantly greater. Further intergroup comparisons revealed that, compared with the PCL (*p* < 0.001), PCL + EGCG (*p* < 0.01), and PCL+CNT (*p* < 0.05) groups, the PCL+EGCG+CNT scaffold resulted in significantly greater nerve volume (Fig. 9B). In addition to volume, the density of regenerated nerve fibers was assessed. Compared with the axotomy group, all the scaffold groups presented significantly greater fiber density: PCL (*p* < 0.05), PCL + EGCG (*p* < 0.01), PCL+CNT (*p* < 0.01), and PCL+EGCG+CNT (*p* < 0.0001). Moreover, the PCL+EGCG+CNT group presented greater fiber density than the PCL (*p* < 0.001), PCL+EGCG (*p* < 0.01), and PCL+CNT (*p* < 0.01) groups did, indicating enhanced regenerative performance. These findings confirm that the synergistic incorporation of EGCG and CNTs into the nanofibrous scaffold significantly improved both the structural volume and fiber density of the repaired sciatic nerve, supporting its potential for advanced peripheral nerve regeneration.

#### Myelin sheath thickness in the sciatic nerve

To further assess the quality of nerve regeneration, histological staining with osmium tetroxide and Luxol Fast Blue (LFB) was performed to visualize myelinated fibers in the regenerated sciatic nerves. Figures 9C and 9E show representative images from each group. Quantitative analysis of myelin sheath thickness revealed that the PCL + EGCG (*p* < 0.05), PCL+CNT (*p* < 0.01), and PCL+EGCG+CNT (*p* < 0.0001) groups presented significantly thicker myelin sheaths than did the axotomy group, indicating enhanced remyelination. Moreover, the PCL+EGCG+CNT group presented markedly greater myelin thickness than both the PCL (*p* < 0.001) and PCL+EGCG (*p* < 0.01) groups did, suggesting a synergistic effect of EGCG and CNTs in promoting myelin regeneration (Fig. 9D–F). These findings confirm that the composite scaffold not only supports axonal regrowth but also facilitates functional remyelination, which is critical for restoring nerve conduction and overall recovery.

#### Immunohistochemical characteristics of the sciatic nerve

**Fig. 10:**
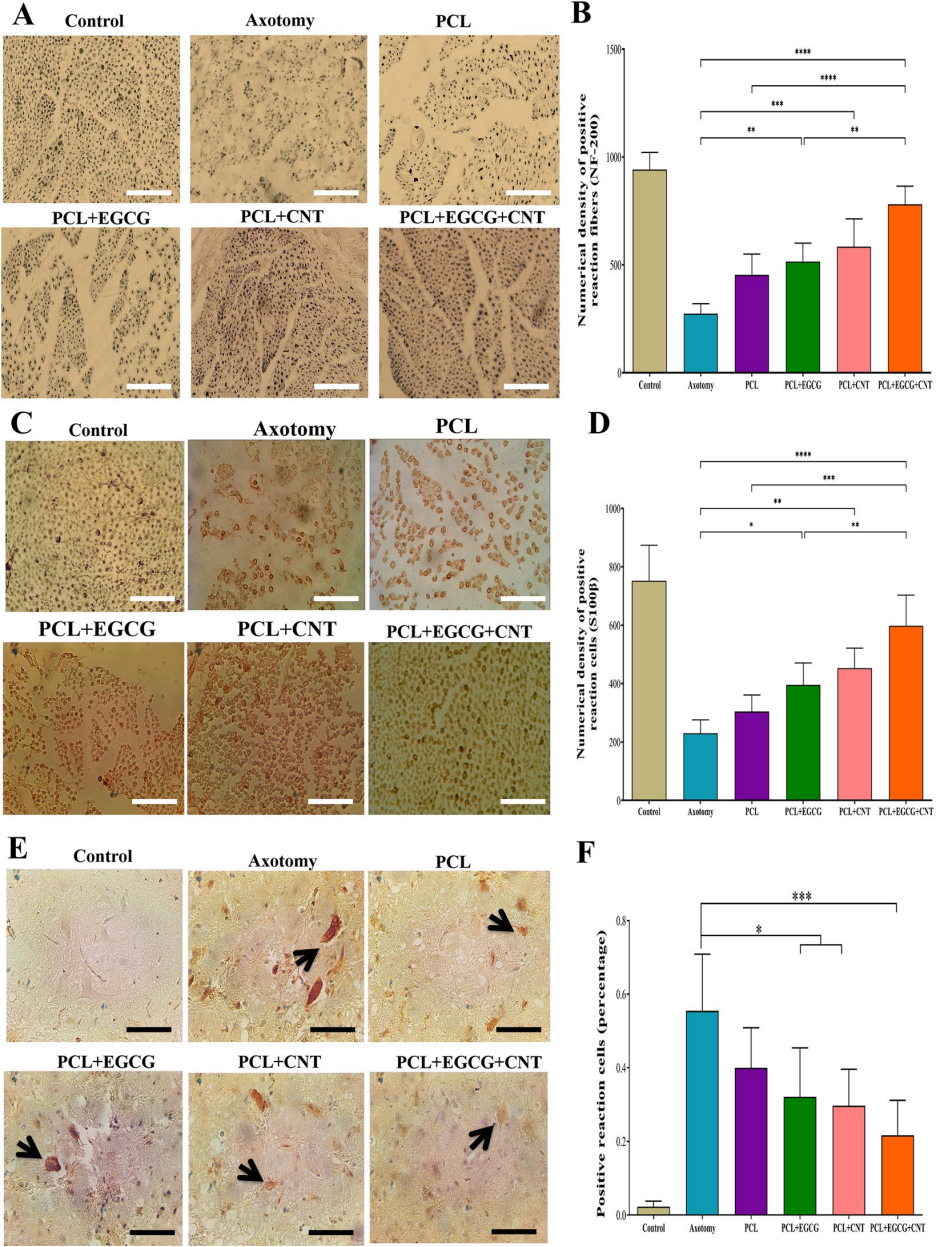
Immunohistochemical evaluation of neural regeneration, Schwann cell activity, and apoptosis following conduit implantation. (**A**) Immunostaining of regenerated sciatic nerve sections with an anti-NF-200 antibody to visualize mature axons (scale bar = 50 µm). (**B**) Quantitative analysis of NF-200-positive nerve fiber density via the optical dissector method. (**C**) Immunostaining of Schwann cells with an S100β antibody (scale bar = 50 µm). (**D**) Quantitative analysis of the S100β-positive Schwann cell density via the optical dissector method. (**E**) Numerical density of glial cells across experimental groups. (**F**) Immunostaining of apoptotic cells in the anterior horn of the spinal cord via an anti-caspase-3 antibody (scale bar = 100 µm) was used to assess the neuroprotective effects of the conduits. The data are presented as the means ± SDs (*n* = 3). Statistical significance is indicated as *p* < 0.05; **p < 0.01, ***p < 0.001, ****p < 0.0001

**Fig. 11:**
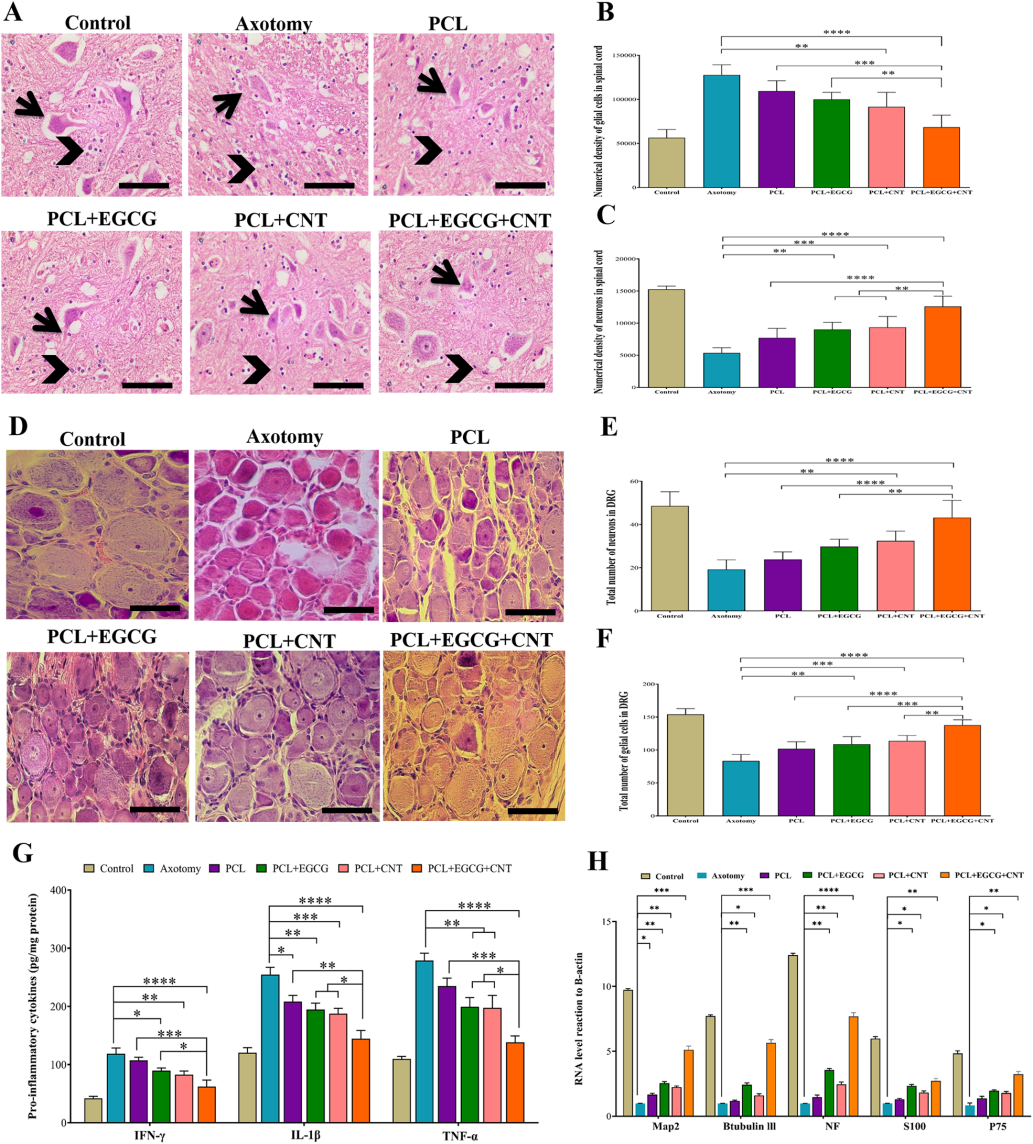
Histological and molecular evaluation of the spinal cord and dorsal root ganglia (DRG) following conduit implantation. (**A**) Representative H&E-stained micrographs of the anterior horn of the spinal cord (arrows: neurons; arrowheads: glial cells) (scale bar = 100 µm). (**B**) Quantitative analysis of neuronal numerical density in the anterior horn. (**C**) Glial cell density in the anterior horn was analyzed via densitometry. (**D**) Representative H&E-stained micrographs of DRGs associated with the injured sciatic nerve (arrows: neurons; arrowheads: glial cells) (scale bar = 100 µm). (**E**) Total neuronal count in the DRG. (**F**) Total glial cell count in the DRG. (**G**) The levels of proinflammatory cytokines (e.g., TNF-α, IL-1β, and IL-6) were measured via ELISA to assess the anti-inflammatory effects of PCL-based scaffolds. (**H**) Comparisons of the mRNA expression levels of Map2, β-tubulin iii, S100, NF, and P75 across the experimental groups were performed via real-time RT‒PCR. The data are presented as the means ± SDs (*n* = 3). Statistical significance is indicated as *p < 0.05, **p < 0.01, ***p < 0.001, and ****p < 0.0001

Images of immunohistochemical staining for NF-200 are presented in Fig. 10A. In the quantitative analysis, the PCL, PCL+EGCG, PCL+CNT, and PCL+EGCG+CNT groups presented significantly greater levels of NF-200-expressing neurons than did the axotomy group (*p* < 0.05, *p* < 0.01, *p* < 0.001, and *p* < 0.0001, respectively). The density of NF-200-positive cells across the various intervention groups was significantly greater in the PCL+EGCG+CNT group than in the PCL, PCL+EGCG, and PCL+CNT groups (*p* < 0.0001, *p* < 0.01, and *p* < 0.05, respectively) (Fig. 11B). Images of samples subjected to immunohistochemical staining for S100β are displayed in Fig. 10C. In the quantitative analysis, the PCL+EGCG, PCL+CNT, and PCL+EGCG+CNT groups presented markedly greater numbers of S100β-positive cells than did the axotomy group (*p* < 0.05, *p* < 0.001, and *p* < 0.0001, respectively). Furthermore, in the analysis of sciatic density across the various experimental groups, the PCL+EGCG+CNT group presented a considerably greater density than the PCL, PCL+EGCG, and PCL+CNT groups did (*p* < 0.001, *p* < 0.01, and *p* < 0.05, respectively). Additionally, the PCL+CNT group had significantly greater numbers of S100β-positive cells than the PCL group did (*p* < 0.05) (Fig. 10D).

#### Immunohistochemical analysis of apoptosis in the spinal cord

Figure 10 Eshows the results of the immunohistochemical analysis of caspase-3. Compared with the axotomy group, the PCL+EGCG, PCL+CNT, and PCL+EGCG+CNT groups presented significant reductions in positive cells, with *p* < 0.05, *p* < 0.05, and *p* < 0.001, respectively (Fig. 11F).

#### Motoneuron and neuroglial cell counts and anterior horn volume analysis in the spinal cord

Figure 11 A shows images of H&E staining of the anterior horn of the spinal cord. A comparison of the neuronal numerical density revealed that the PCL, PCL+EGCG, PCL+CNT, and PCL+EGCG+CNT groups presented significantly greater counts of neurons than did the axotomy group (*p* < 0.05, *p* < 0.01, *p* < 0.001, and *p* < 0.0001, respectively). Moreover, the PCL+EGCG+CNT group presented a notably greater number of neurons than the PCL, PCL+EGCG, and PCL+CNT groups did (*p* < 0.0001, *p* < 0.01, and *p* < 0.01, respectively) (Fig. 11B). Compared with the axotomy group, the PCL+EGCG, PCL+CNT, and PCL+EGCG+CNT groups presented substantially fewer glial cells (*p* < 0.05, *p* < 0.01, and *p* < 0.0001, respectively). Additionally, the PCL+EGCG+CNT experimental groups presented significantly fewer glial cells than the PCL, PCL+EGCG, and PCL+CNT groups did (*p* < 0.001, *p* < 0.01, and *p* < 0.05, respectively) (Fig. 11C).

#### Number and distribution of neurons and satellite cells in the DRG

Histological evaluation of dorsal root ganglia (DRGs) via hematoxylin and eosin (H&E) staining was performed to assess neuronal and glial cell responses following sciatic nerve injury and scaffold implantation. Figure 11Dpresents representative micrographs of DRG architecture across the experimental groups. Quantitative analysis of neuronal density revealed significantly greater cell counts in the PCL+EGCG (*p* < 0.05), PCL+CNT (*p* < 0.01), and PCL+EGCG+CNT (*p* < 0.0001) groups than in the axotomy group. Furthermore, the PCL+EGCG+CNT group presented markedly greater neuronal density than the PCL (*p* < 0.0001), PCL+EGCG (*p* < 0.01), and PCL+CNT (*p* < 0.05) groups did, indicating enhanced neuroprotective and regenerative effects (Fig. 11E). In parallel, glial cell density within the DRG was assessed. Compared with the axotomy group, the PCL (*p* < 0.05), PCL+EGCG (*p* < 0.01), PCL+CNT (*p* < 0.001), and PCL+EGCG+CNT (*p* < 0.0001) groups presented significantly greater glial cell counts, reflecting active neuroglial support. Notably, the PCL+EGCG+CNT group presented significantly greater glial density than the PCL (*p* < 0.0001), PCL+EGCG (*p* < 0.001), and PCL+CNT (*p* < 0.05) groups did (Fig. 11F), suggesting synergistic enhancement of glial-mediated repair mechanisms. The composite scaffold not only promotes neuronal survival but also stimulates glial cell activity within the DRG, contributing to a more robust regenerative microenvironment.

#### Assessment of proinflammatory mediators in the sciatic nerve

To evaluate the inflammatory response following sciatic nerve injury and scaffold implantation, the levels of proinflammatory cytokines—including IFN-γ, IL-1β, and TNF-α—were quantified via ELISA. Figure 11 Gpresents the comparative cytokine profiles across the experimental groups.

Compared with the axotomy group, the PCL+EGCG (*p* < 0.05), PCL+CNT (*p* < 0.01), and PCL+EGCG+CNT (*p* < 0.0001) groups presented significantly lower levels of IFN-γ. Similarly, the IL-1β levels were significantly lower in the PCL+EGCG (*p* < 0.01), PCL+CNT (*p* < 0.001), and PCL+EGCG+CNT (*p* < 0.0001) groups. The TNF-α levels also decreased significantly in the same groups (*p* < 0.01, *p* < 0.01, and *p* < 0.0001, respectively). Notably, compared with the axotomy group, the PCL group presented significantly lower IL-1β levels (*p* < 0.01), but not significantly lower IFN-γ or TNF-α levels. Further intergroup comparisons revealed that the PCL+EGCG+CNT group presented significantly lower levels of IFN-γ (*p* < 0.001 vs PCL; *p* < 0.05 vs PCL+EGCG), IL-1β (*p* < 0.01 vs PCL; *p* < 0.05 vs PCL+EGCG), and TNF-α (*p* < 0.001 vs PCL; *p* < 0.05 vs PCL+EGCG). Additionally, the IL-1β and TNF-α levels were significantly lower in the PCL+EGCG+CNT group than in the PCL+CNT group (both *p* < 0.05). The synergistic incorporation of EGCG and CNTs into the PCL scaffold effectively attenuated the inflammatory response, contributing to a more favorable microenvironment for nerve regeneration.

#### Analysis of Map2, βIII-Tubulin, neurofilament, S100, and p75NTR expression in sciatic nerve regeneration

To investigate the molecular mechanisms underlying nerve regeneration, gene expression analysis was performed on sciatic nerve tissue 12 weeks after nerve guidance conduit (NGC) implantation. The mRNA levels of neuronal markers—Map2, βIII-tubulin, and neurofilament (NF)—as well as Schwann cell markers—P75 and S100—were quantified via real-time RT‒PCR. As shown in Fig. 11H, the PCL+EGCG+CNT and PCL+EGCG groups presented significantly elevated expression levels of all five markers compared with those of the axotomy group (*p* < 0.01 and *p* < 0.05, respectively), indicating increased neuronal and glial activation. Furthermore, the PCL+EGCG+CNT group presented significantly greater expression of these genes than the PCL, PCL+EGCG, and PCL+CNT groups did (*p* < 0.05), suggesting a synergistic effect of EGCG and CNTs in promoting both axonal regeneration and Schwann cell support. These molecular findings corroborate the histological and functional outcomes, reinforcing the superior regenerative potential of the PCL+EGCG+CNT composite scaffold.

### Functionality assay

**Fig. 12:**
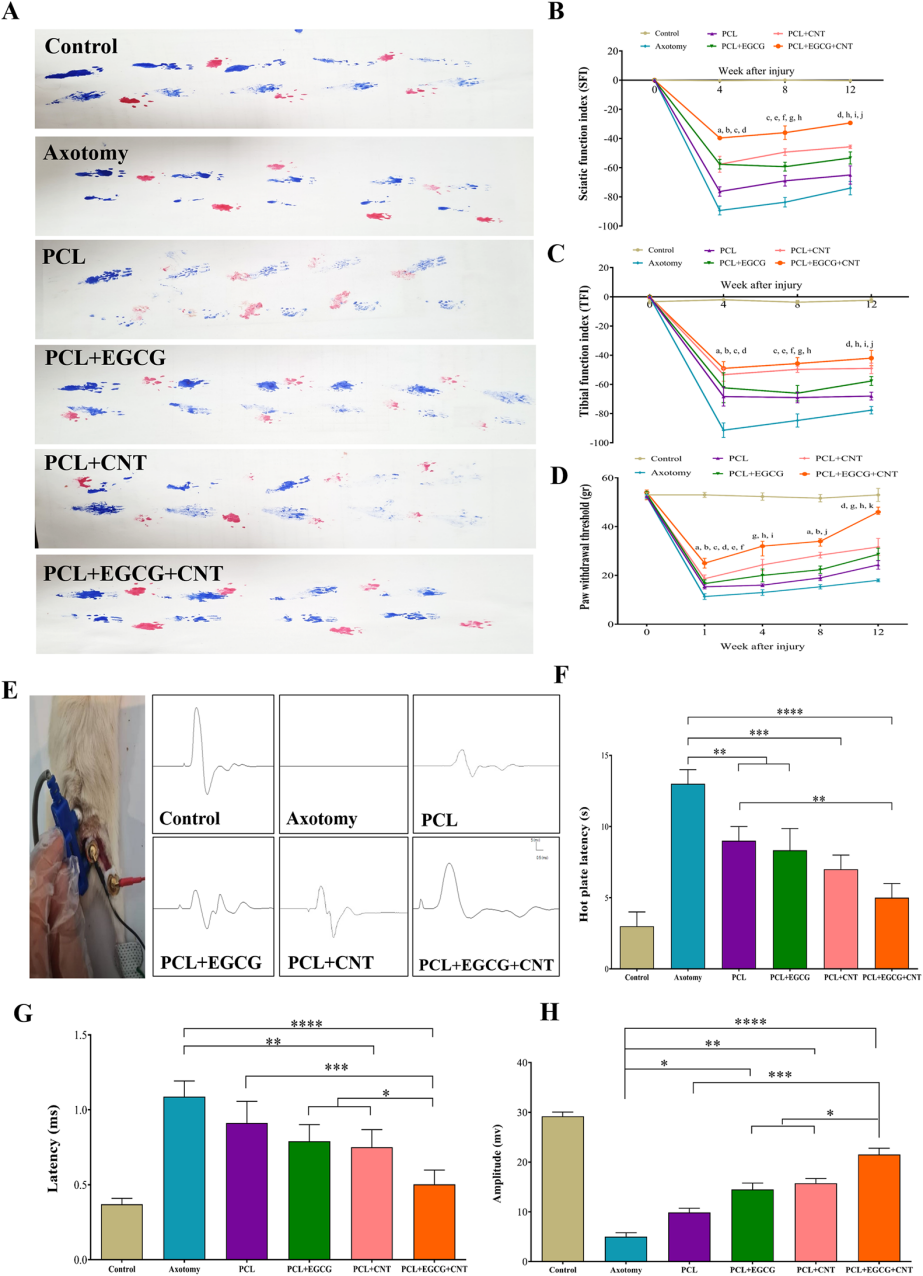
The impact of PCL loaded with EGCG and CNTs on the sciatic functional index. (**A**) Pawprints of rats in the sciatic functional index (SFI) test. (**B**-**C**) SFI and TFI tests were performed before and at 4, 8, and 12 weeks after injury. ^a^ *p* < 0.05 PCL and PCL+EGCG groups vs axotomy group; ^b^ *p* < 0.01 PCL+CNT and PCL+EGCG+CNT groups vs axotomy group; ^c^ *p* < 0.05 PCL+CNT and PCL+EGCG+CNT groups vs PCL group; ^d^ *p* < 0.05 PCL+EGCG+CNT group vs PCL+EGCG group; ^e^ *p* < 0.05 PCL group vs axotomy group; ^f^ *p* < 0.01 PCL+EGCG, PCL+CNT, and PCL+EGCG+CNT groups vs axotomy group; ^g^ *p* < 0.01 PCL+EGCG group vs PCL group; ^h^ *p* < 0.05 PCL+EGCG+CNT group vs PCL+CNT group; ^i^ *p* < 0.05 PCL+CNT and PCL+EGCG+CNT groups vs axotomy group; ^j^ *p* < 0.01 PCL+EGCG+CNT group vs PCL+CNT group. The impact of PCL loaded with EGCG and CNTs on von Frey filaments. (**D**) Von Frey filament tests were performed before and at 1, 4, 8, and 12 weeks after injury. ^a^ *p* < 0.05 PCL and PCL+EGCG groups vs axotomy group; ^b^ *p* < 0.01 PCL+CNT group vs axotomy group; ^c^ *p* < 0.001 PCL+EGCG+CNT group vs axotomy group; ^d^ *p* < 0.05 PCL+EGCG+CNT group vs PCL+EGCG and PCL+CNT groups; ^e^ *p* < 0.05 PCL+EGCG group vs PCL group; ^f^ *p* < 0.01 PCL+CNT group vs PCL group; ^g^ *p* < 0.05 PCL+EGCG and PCL+CNT group vs axotomy group; ^h^ *p* < 0.01 PCL+EGCG+CNT group vs axotomy group; ^i^ *p* < 0.05 PCL+EGCG+CNT group vs PCL, PCL+EGCG, and PCL+CNT groups; ^j^ *p* < 0.01 PCL+EGCG+CNT group vs axotomy group; ^k^ *p* < 0.05 PCL+EGCG+CNT group vs PCL group. Muscle activity in rats. (**E**) The sciatic nerve was stimulated, and the muscle action potential in the gastrocnemius muscle was recorded. (**F**) Hot plate latency at week 12 after injury (second; S). (**H**) the impact of PCL loaded with EGCG and CNTs on EMG latency. EMG latency at week 12 after injury (Millisecond; MS). (**G**) Amplitude was measured in all groups. Asterisks indicate differences between groups *p < 0.05; **p < 0.01, ***p < 0.001, ****p < 0.0001. The impact of PCL loaded with EGCG and CNTs on hot plate latency

In the present study, the hot plate latency, von Frey filaments, EMG latency, NCV, SFI, and TFI were investigated to assess neurological functions. Fig. 12A shows the paw prints of the rats in the SFI test. The SFI test results revealed that the PCL, PCL+EGCG, PCL+CNT, and PCL+EGCG+CNT groups presented significantly higher scores than did the axotomy group at week 4 (*p* < 0.05, *p* < 0.05, *p* < 0.01, and *p* < 0.01, respectively) and week 8 (all *p* < 0.05). At week 12, only the PCL+CNT and PCL+EGCG+CNT groups presented significantly elevated scores in comparison with those of the axotomy group (*p* < 0.05 and *p* < 0.01, respectively). In addition, the PCL+EGCG+CNT group presented significantly higher scores at week 4 than did the PCL group (*p* < 0.05), at week 8 compared with both the PCL and PCL+CNT groups (both *p* < 0.05), and at week 12, the PCL, PCL+CNT, and PCL+EGCG groups presented higher scores than did the PCL, PCL+CNT, and PCL+EGCG groups did (*p* < 0.01, *p* < 0.05, and *p* < 0.05, respectively). Moreover, the PCL+CNT mat presented considerably greater scores than did the PCL mat at weeks 4 and 8 (both *p* < 0.05). Moreover, the PCL+EGCG group achieved notably higher scores than did the PCL group at week 8 (*p* < 0.01) (Fig. 12B). In the TFI test, the PCL, PCL+EGCG, PCL+CNT, and PCL+EGCG+CNT groups had significantly higher scores than did the axotomy group at weeks 4 and 8 (all *p* < 0.05). At week 12, only the PCL+CNT and PCL+EGCG+CNT groups presented significantly elevated scores compared with those of the axotomy group (*p* < 0.05 and *p* < 0.01, respectively). Additionally, the PCL+EGCG+CNT group presented significantly higher scores at week 4 than did the PCL group did (*p* < 0.05), at week 8 compared with both the PCL and PCL+CNT groups (both *p* < 0.05), and at week 12, the PCL, PCL+CNT, and PCL+EGCG groups presented significantly higher scores compared to the PCL, PCL+CNT, and PCL+EGCG groups (*p* < 0.01, *p* < 0.05, and *p* < 0.05, respectively). Furthermore, the PCL+CNT group had significantly higher scores than did the PCL group at weeks 4 and 8 (both *p* < 0.05), whereas the PCL+EGCG group achieved notably higher scores than did the PCL group at week 8 (*p* < 0.01) (Fig. 12C). With respect to the von Frey filaments test, the PCL+EGCG, PCL+CNT, and PCL+EGCG+CNT groups presented substantially higher scores than did the axotomy group during weeks 1 (*p* < 0.05, *p* < 0.01, and *p* < 0.001, respectively), 4 (*p* < 0.05, *p* < 0.05, and *p* < 0.01, respectively), 8 (*p* < 0.05, *p* < 0.01, and *p* < 0.01, respectively), and 12 (*p* < 0.05, *p* < 0.05, and *p* < 0.01, respectively). Additionally, the PCL group had substantially higher scores than did the axotomy group during week 1 (*p* < 0.05). Moreover, when the von Frey filament test scores across the various experimental groups were assessed, the PCL+EGCG+CNT group presented substantially higher scores than did the PCL+EGCG and PCL+CNT groups during weeks 1 and 12 (both groups, *p* < 0.05) and higher scores than did the PCL group during weeks 2 and 12 (both groups, *p* < 0.05). Furthermore, the PCL+EGCG group presented markedly higher scores than did the PCL group at week 1 (*p* < 0.05) (Fig. 12D). Figure 12E Shows that in the EMG and NCV latency assessments, the reaction time of the muscle response to nerve activation was markedly lower in the PCL+EGCG, PCL+CNT, and PCL+EGCG+CNT groups than in the axotomy group (*p* < 0.05, *p* < 0.01, and *p* < 0.0001, respectively). Moreover, the PCL+EGCG+CNT group exhibited notably reduced latency compared with the PCL, PCL+EGCG, and PCL+CNT groups. (*p* < 0.001, *p* < 0.05, and *p* < 0.05, respectively) (Fig. 12F).

The NCV amplitude recorded in the gastrocnemius muscles was significantly greater following treatment with the PCL+EGCG+CNT scaffold than following treatment with the PCL, PCL+EGCG, or PCL+CNT scaffold (*p* value < 0.05) (Fig. 12 H). In the hot plate latency assessment, the PCL, PCL+EGCG, PCL+CNT, and PCL+EGCG+CNT groups presented markedly lower scores than did the axotomy group (*p* < 0.01, *p* < 0.01, *p* < 0.001, and *p* < 0.0001, respectively). In addition, the PCL+EGCG+CNT group presented markedly lower scores than the PCL and PCL+EGCG groups did (*p* < 0.01 and *p* < 0.05, respectively) (Fig. 12G).

## Discussion

The tissue engineering field is a pivotal area within regenerative medicine, offering innovative methodologies to enhance tissue repair and optimize therapeutic results. This interdisciplinary technique combines principles from biology, engineering, and materials science to develop scaffolds and constructs that can regenerate or replace damaged tissues and organs [62–64]. Although allografts and autografts are potential treatments for traumatic nerve injuries, their limitations necessitate the development of fully degradable NGCs with suitable mechanical, physical, and biological properties to advance sciatic nerve regeneration [65]. Electrospun nanofibrous conduits have attracted much attention. Owing to their nanoscale fiber diameters, these fibers aim to replicate the natural extracellular matrix, facilitating optimal nerve repair. Recent research has shown promising outcomes for peripheral nerve regeneration, with ongoing investigations exploring their applications within the CNS [66].

Because of its biodegradability and biocompatibility, PCL is a favorable candidate for NGC development [67]. The CNTs that combine with PCL enhance the mechanical and physicochemical features of the conduits, which is essential for sciatic nerve regeneration [68, 69]. EGCG stimulates an Nrf2-dependent antioxidant response, protecting cells from oxidative stress. Its inclusion promotes nerve regeneration and reduces inflammation in peripheral nerve injuries [70–72].

The structured fibrous composition emulates native tissue, providing mechanical stimuli that control cellular function. The electrospun PCL+EGCG+CNT nanofibrous conduits support cell proliferation and provide physical signals that influence cellular activities. Duan et al. reported that adding CNTs to PLLA-based nanofibers enhanced the electrical conductivity [73]. Similarly, in the present study, the incorporation of CNTs into PCL tubes increased the hydrophilicity and tensile strength. Furthermore, our results revealed a reduction in fiber diameter, which is consistent with previous findings [74]. Composites containing EGCG and nanocarbons exhibit dynamic electrical behavior. A study reported that the presence of EGCG in SWNT/EGCG films decreased the conductivity with increasing interlayer spacing, but by adding H₂O₂ – oxidant, electron transport was restored and the conductivity performance was enhanced [75]. Additionally multiwalled nanocarbons can also be modified with a thin layer of a mixed matrix (silanized MXene) that is conductive and antibacterial [76]. On the other hand, EGCG causes the dispersion of nanotubes, causing the CNTs to be nonagglomerated and to be arranged in discrete or regular networks in the matrix. This order in the dispersion helps to increase the continuous paths for electrical charge transfer, thus improving the electrical conductivity of the composite [77]. CNTs also have enhanced conduit elasticity, facilitating mechanical adaptability and structural resilience and their role in functional nerve regeneration remains secondary to biochemical modulation [78]. Since inflammation is a critical factor affecting axonal regeneration, cell viability, and overall functional recovery, EGCG reduces fibrosis through its anti-inflammatory effect and improves the cellular microenvironment by increasing Schwann cell activity, thereby promoting nerve injury recovery [79].

Elasticity is critical for canal function, but controlling inflammation should be a therapeutic priority to ensure long-term regeneration and function. AFM studies revealed that the addition of EGCG and CNTs to PCL fibers increased surface roughness and promoted cellular differentiation [80, 81]. The pH of the samples containing CNTs was most similar to that of the samples containing PBS (pH = 7.4). CNTs potentially help reduce inflammatory responses in acidic environments by reducing and modulating the pH, in accordance with Díaz E et al. (Fig. 2A–B) [82]. Although the slight decrease in pH remained within a range that is generally safe for cell survival and nerve regeneration, it is important to examine how the microenvironment evolves over the long term. Future long-term studies are needed to understand whether such changes could affect scaffold function or the regeneration process [83].

The mechanical properties of nerve regeneration conduits require constructs with minimal swelling ratios and gradual degradation rates. The degradation time allows axonal growth in the injured area by controlling cell proliferation and repair (Fig. 2A, D) [84, 85]. Consistent with previous studies, SEM images and MTT assays revealed normal cell viability and a spindle-shaped morphology when the cells were grown on PCL+EGCG+CNT conduits after 7 days. CNTs promote cell adhesion, proliferation, and maturation via efficient electrical signal transmission [25, 73]. The structural integrity of conduits is crucial, as inadequate strength can lead to failure post-transplantation or tearing during suturing. This study demonstrated that in EGCG+CNT+PCL/collagen composite conduits, the PCL and CNT polymers provide conduit strength, and EGCG and collagen act as bioactive modulators by forming a regenerative microenvironment that provides biochemical signaling [19, 86, 87].

The structural integrity of the tubes is crucial, as insufficient strength can lead to failure after grafting or tearing during suturing. In this study, the findings showed that the incorporation of CNTs into nanofibers changes their surface and physical properties. [73, 81, 88] Next, to maintain the properties of the extracellular matrix, we filled the hollow PCL+EGCG+CNT tubes with a collagen hydrogel. This leads to increased cell adhesion and axon growth [41].

The present study investigated the effects of EGCG on neural conduits and revealed that the levels of inflammatory factors were reduced. This reduction, together with the CNT conductivity and PCL mechanics, facilitated the formation of new nerve fibers, as demonstrated by IHC and H&E staining (Figs. 8 and 9). On the other hand, satellite glial cells in the dorsal root ganglion (DRG) exhibit stem cell-like properties. Consistent with previous studies, the results of this study revealed that in the groups treated with the PCL+EGCG+CNT conduit, neural pathways increased activity and neuronal regeneration compared with those in the injured group (Fig. 9A–B) [89, 90].

This study revealed increased expression of the MAP2, β-tubulin III, and NF genes in the PCL+EGCG+CNT and PCL+CNT groups compared with the control group. NF is a key regulator of neuronal growth, plasticity, and maintenance. The molecular changes observed in this study are consistent with those reported in existing studies and suggest a synergistic role of cytoskeletal proteins, neurotrophins, and growth factors in supporting nerve regeneration. While these observations contribute to the understanding of the cellular mechanisms of repair, further studies are needed to validate their long-term functional relevance and translational potential (Fig. 10) [91–93].

In the present study, an increase in the S100 gene was observed in the PCL+EGCG+CNT group. S100 proteins are produced in the cytoplasm of Schwann cells and act as markers in the peripheral nervous system. S100 regulates protein phosphorylation, transcription factor activity, the calcium ion (Ca^2 +^) balance, cytoskeletal dynamics, enzyme functions, cell proliferation, specialization, and various cellular responses [94, 95]. EGCG may contribute to Schwann cell proliferation, the modulation of oxidative stress, and the suppression of inflammatory cytokines, thereby supporting axonal regrowth [86].

Our findings revealed increased expression of both S100 and NF-200, which are indicative of neural development and structural maturation. These results are consistent with those of previous studies, although other factors, such as conduit architecture, degradation rate, and host-specific responses, may also influence gene expression. S100β, a specific isoform expressed by differentiated Schwann cells, has been reported to facilitate their migration along neovascular structures and promote further differentiation. The presence of NF-200, a neuronal cytoskeletal marker, is associated with enhanced axonal integrity and motor function recovery [96]. While these molecular changes suggest a regenerative response, rodent models may not fully recapitulate the complexity of human nerve repair, particularly in terms of scale, immune dynamics, and long-term functional outcomes. Further studies are needed to validate these findings in clinically relevant models and explore the sustained impact of EGCG and CNTs on peripheral nerve regeneration [97]. The combination of EGCG and CNTs within PCL conduits was associated with the formation of thicker myelin sheaths, suggesting a potential synergistic effect on myelination. EGCG possesses a tetracyclic structure with eight hydroxyl groups, contributing to its hydrophilicity and antioxidant activity [98]. The majority of the bioactivities of EGCG are attributed to its antioxidant properties. EGCG has been shown to protect against nerve damage by inhibiting apoptosis, reducing reactive oxygen species (ROS), and increasing neurofilament light chain (NF-L) expression following injury [99]. The successful formation of newly myelinated neurons in the PCL+EGCG+CNT group is consistent with previous studies demonstrating the potential of EGCG and CNTs. CNTs may support neuronal growth and differentiation by improving the mechanical and electrical properties of the scaffold. However, other factors—such as scaffold architecture, degradation kinetics, and the host response—may also contribute to these results [100]. Previous studies have shown that biomaterials with hemolysis ratios less than 5% are considered highly biocompatible [101]. The PCL+EGCG+CNT group had a 4.27% success rate, confirming its suitability for biomedical applications. The considerably lower IFN-γ, IL-1β, and TNF-α levels in the PCL+EGCG+CNT group indicate the synergistic effects of EGCG and CNTs. Modified, multiparametric NGCs can facilitate axonal repair by reducing the levels of inflammatory factors [102]. This evidence, confirming previous research, suggests that combining bioactive molecules with conductive materials can amplify therapy by reducing inflammation and promoting repair [70, 103, 104]. The present study focused primarily on the analysis of inflammatory cytokines (TNF-α, IL-1β, and IL-6), which indicate the inflammatory to reparative transition after nerve injury. However, direct detection of macrophage polarization (e.g., CD68+CD163+ for M2, CD68+CD163- for M1) would strengthen our findings of immune modulation by confirming this change at the cellular level [105, 106]. Future studies, if they include the evaluation of macrophage markers (CD68, CD163) and neutrophil markers (MPO), would provide a more comprehensive understanding of the spatiotemporal inflammatory and reparative processes occurring around the implanted conduit. [107]. If inflammation persists, astrocytes become hyperactivated and secrete inhibitory proteoglycans (CSPGs) to form glial scars, which act as a physical and chemical barrier to axonal regrowth and neuronal repair. EGCG inhibits the secretion of inflammatory cytokines (TNF-α, IL-1β, and IL-6) by inhibiting the NF-κB and MAPK pathways (long noncoding RNAs) to mediate cerebrovascular injury after CNS injury [108]. Our results suggest that combining bioactive molecules (e.g., EGCG) with conductive materials (e.g., CNTs or conductive polymers) can both reduce inflammation and enhance tissue repair. However, despite these results, further studies are still needed to confirm and validate these effects in more severe or realistic nerve injury models (e.g., with large distances between nerve endings or in human models). Our study revealed that the PCL+EGCG+CNT scaffold provided good functional motor recovery and outperformed the other groups in terms of the TFI. Walking pattern analysis is a widely used method for assessing neuromuscular recovery after sciatic nerve injury [109]. Our results revealed that, compared with the other groups, the PCL+EGCG+CNT scaffold group presented improved motor function, and the SFI and TFI values were significantly greater after three months. These findings suggest that the combined effects of EGCG and CNTs may contribute to enhancing sciatic nerve recovery, although other factors, such as scaffold architecture and the inherent characteristics of each organism, may also play a role (Fig. 11B–C) [110]. The functional improvement observed in the PCL+EGCG+CNT group suggested an interaction between the conductive network of CNTs and the bioactive effects of EGCG. The prolonged pain response in the hot plate test is indicative of a reduction in neuropathic sensitivity, which has been reported in previous studies to be due to the anti-inflammatory and neuroprotective properties of EGCG. In addition, the preservation of gastrocnemius muscle mass and the increase in skeletal muscle fiber regeneration indicate that this composite scaffold also promoted neuromuscular recovery. These findings are related to the ability of EGCG to reduce lactate accumulation and induce a more oxidative muscle phenotype, ultimately indicating functional improvement after nerve injury [111]. In addition, previous studies have shown that EGCG supplementation can attenuate muscle loss in sarcopenia by inhibiting the ubiquitin–proteasome pathway, thereby reducing protein degradation and enhancing anabolic signaling. While these findings support the potential of EGCG in neuromuscular recovery, further investigation is needed to confirm its long-term efficacy and translational relevance in larger animal models and clinical settings [112]. Twelve weeks after surgery, EMG assessments confirmed the functional improvement achieved by the PCL+EGCG+CNT scaffold. Compared with those of the control and single-component groups, the improved EMG and NCV parameters indicate that axonal regeneration and repair occurred. These findings are consistent with previous studies showing that conductive nanocomposites facilitate nerve signal transmission, thus accelerating Schwann cell activity in peripheral nerve repair. These results can also be attributed to the anti-inflammatory effects of EGCG. During electrospinning, EGCG molecules are physically entrapped in the PCL nanofibers, allowing their gradual release and sustained local bioactivity. This controlled transport likely contributes to the microenvironment of nerve regeneration. However, further studies using larger nerve injury models or more complex organisms are needed to evaluate long-term functional outcomes [113]. The hydrophobic properties of PCL polymers contribute to the gradual release of EGCG, thereby maintaining prolonged antioxidant activity. In addition, EGCG forms π and hydrogen bonds with the surface of CNTs, enhancing their adsorption. This interaction can also increase the electrical conductivity of the scaffold. In addition, the collagen hydrogel acts as a secondary site and stabilizes EGCG through hydrogen bonding and electrostatic interactions [114]. Thus, it mimics the matrix, which facilitates cell adhesion and migration and EGCG release. Collectively, these synergistic mechanisms enhance the biological activities of EGCG, reduce oxidative stress and inflammation, and promote neuronal maturation and axonal regrowth [115]. While the results are encouraging, to transfer this platform to clinical settings, the complexity of multiple factors—including the rate of scaffold degradation, host immune response, and anatomical variability—that may affect functional outcomes must be evaluated.

## Conclusion

This work shows that electrospun scaffolds prepared from PCL, EGCG, and CNTs can create a supportive environment for nerve regeneration. By combining the antioxidant and bioactive properties of EGCG with the conductivity of CNTs, the composite scaffold provided better mechanical strength, smaller fiber diameters, and a more hydrophilic surface than PCL alone. These features help cells attach and proliferate in vitro, and in animal experiments, they are associated with improved expression of neuronal and glial markers as well as improved functional recovery after sciatic nerve injury. These changes were accompanied by improved motor function and enhanced electrophysiological parameters. For this approach to be clinically applicable in the future, long-term safety and efficacy evaluations in large animal models are needed. Regulatory approval also depends on compliance with equipment standards, including GMP-based scaffold manufacturing and validation.

## Data Availability

The data are available from the authors upon reasonable request and with the permission of the corresponding authors.

## Abbreviations

AFM: Atomic Force Microscopy
CO₂: Carbon Dioxide
COOH: Carboxyl Group
CNTs: Carbon nanotubes
CNT-COOH: Carbon Nanotubes Functionalized with Carboxyl Groups
DAB: Diaminobenzidine
DAPI: 4′, 6-diamidino-2-phenylindole
DMEM/F12: Dulbecco’s modified Eagle’s medium
DMSO: Dimethyl sulfoxide
DRG: Dorsal root ganglion
EDTA: Ethylenediaminetetraacetic acid
EGCG: Epigallocatechin gallate
ELISA: Enzyme-Linked Immunosorbent Assay
EMG: Electromyography
FBS: Fetal bovine serum
FDA: Food and Drug Administration
FESEM: Field Emission Scanning Electron Microscopy
FOV: Field of View
FTIR: Fourier transform infrared spectroscopy
FTIR-ATR: Fourier transform infrared – attenuated total reflectance
H&E: Hematoxylin and Eosin
HPL: Hot plate batency
IFN-γ: Interferon Gamma
IL-1β: Interleukin-1 Beta
ISO: International Organization for Standardization
LFB: Luxol fast blue
MAP2: Microtubule-Associated Protein 2
MRI: Magnetic Resonance Imaging
MSCs: Mesenchymal Stem Cells
MTT: 3-(4, 5-Dimethylthiazol-2-yl)-2,5- diphenyl tetrazolium bromide
MWCNTs: Multiwall Carbon Nanotubes
NCV: nerve conduction velocity
NF: Neurofilament
NF-200: Neurofilament 200
NGCs: Nerve guidance conduits
NEX: Number of Excitations
PCL: Poly (ε-caprolactone)
PBS: Phosphate-buffered saline
PLA: Polylactic acid
PLGA: Poly (lactic-co-glycolic acid)
PU: Polyurethane
PWTs: Paw withdrawal thresholds
REST: Relative expression software tool
RT-PCR: real-time polymerase chain reaction
SD: Standard Deviation
SFI: sciatic function index
SEM: Scanning electron microscopy
TCP: Tissue Culture Plates
TFI: Tibial functional index
TNF-α: Tumor necrosis factor alpha
TR: repetition time
